# Regulation of the Bone Vascular Network is Sexually Dimorphic

**DOI:** 10.1002/jbmr.3825

**Published:** 2019-10-09

**Authors:** Alice Goring, Aikta Sharma, Behzad Javaheri, Rosanna CG Smith, Janos M Kanczler, Alan Boyde, Eric Hesse, Sumeet Mahajan, Bjorn R Olsen, Andrew A Pitsillides, Philipp Schneider, Richard OC Oreffo, Claire E Clarkin

**Affiliations:** ^1^ School of Biological Sciences University of Southampton Southampton UK; ^2^ Department of Comparative Biomedical Sciences The Royal Veterinary College London UK; ^3^ Bone and Joint Research Group, Centre for Human Development, Stem Cell and Regeneration, Human Development and Health, Institute of Developmental Sciences University of Southampton Southampton UK; ^4^ Dental Physical Sciences, Barts and The London School of Medicine and Dentistry Queen Mary University of London London UK; ^5^ Institute of Molecular Musculoskeletal Research Faculty of Medicine, LMU Munich Planegg‐Martinsried Germany; ^6^ Institute for Life Sciences and Department of Chemistry University of Southampton Southampton UK; ^7^ Department of Developmental Biology Harvard School of Dental Medicine Boston MA USA; ^8^ Bioengineering Research Group University of Southampton Southampton UK

**Keywords:** OSTEOBLASTS, MATRIX MINERALIZATION, BONE QCT/MICROCT, GENETIC ANIMAL MODELS, PRECLINICAL STUDIES

## Abstract

Osteoblast (OB) lineage cells are an important source of vascular endothelial growth factor (VEGF), which is critical for bone growth and repair. During bone development, pubertal differences in males and females exist, but little is known about whether VEGF signaling contributes to skeletal sexual dimorphism. We have found that in mice, conditional disruption of VEGF in osteocalcin‐expressing cells (OcnVEGFKO) exerts a divergent influence on morphological, cellular, and whole bone properties between sexes. Furthermore, we describe an underlying sexual divergence in VEGF signaling in OB cultures in vitro independent of circulating sex hormones. High‐resolution synchrotron computed tomography and backscattered scanning electron microscopy revealed, in males, extensive unmineralized osteoid encasing enlarged blood vessel canals and osteocyte lacunae in cortical bone after VEGF deletion, which contributed to increased porosity. VEGF was deleted in male and female long bone–derived OBs (OBVEGKO) in vitro and Raman spectroscopic analyses of mineral and matrix repertoires highlighted differences between male and female OBVEGFKO cells, with increased immature phosphate species prevalent in male OBVEGFKO cultures versus wild type (WT). Further sexual dimorphism was observed in bone marrow endothelial cell gene expression in vitro after VEGF deletion and in sclerostin protein expression, which was increased in male OcnVEGFKO bones versus WT. The impact of altered OB matrix composition after VEGF deletion on whole bone geometry was assessed between sexes, although significant differences between OcnVEGFKO and WT were identified only in females. Our results suggest that bone‐derived VEGF regulates matrix mineralization and vascularization distinctly in males and females, which results in divergent physical bone traits.

## Introduction

Vascular endothelial growth factor (VEGF) is an endothelial cell (EC) survival factor and plays a central role in the coupling of angiogenesis and osteogenesis.^1^, ^2^ Bone‐forming osteoblast (OB) cells are a predominant source of VEGF in bone and produce VEGF in response to a range of factors, including hypoxia, mechanical strain, and estrogen.^3^, ^4^ Although autocrine and/or direct effects of VEGF on OB differentiation remain disputed in the literature,^3^ intracrine VEGF signaling has been reported and linked to early osteoblast lineage commitment.^1^, ^5^ Today, osteoblast and vascular endothelial cells are known to be heterogeneous in developing bone, with immature and mature osteoblast populations interacting distinctly with the bone vasculature.^6^ In addition to its importance in maintenance of bone homeostasis,^1^ pre‐osteoblast‐derived VEGF has demonstrated to be critical in the process of fracture repair.^4^, ^7^ Furthermore, the vasculature appears to play a contributing role in age‐related skeletal pathologies, and it has been shown that circulating VEGF levels in postmenopausal women are reduced.^8^

There is an acknowledged divergence in age‐related fracture risk between men and women, which is thought to reflect inherent differences in the control of bone growth and development experienced between the onset of puberty and young adulthood. During development, changes in bone geometry are observed to be greater in males than females.^7^, ^9^, ^10^, ^11^ Males produce a larger skeleton, which is postulated to protect them from fracture in later life^11^, ^12^, ^13^, ^14^; however, the underlying mechanisms regulating mineralization during skeletal sexual dimorphism remain poorly understood. Estrogen and androgen therapy have longstanding positive effects on bone mass, but their efficacy is limited in cases of severe osteoporosis.^15^ Thus, identifying whether genetic mechanisms exist and contribute to osteoporosis is of growing importance.

Given the pivotal role of VEGF in coupling osteogenesis and angiogenesis, the aim of this study was to improve our understanding of VEGF signaling in the context of skeletal sexual dimorphism. Herein, we have compared the effects of osteocalcin‐specific VEGF deletion in regulating male and female osteoblast matrix production, mineralization, and linked this to whole bone phenotypes.

## Materials and Methods

### Animal derivation and genotyping

Second‐generation osteocalcin‐specific VEGF knockout mice (OcnVEGFKO) were produced by mating first‐generation VEGF^fl/fl^ mice with a single allele of osteocalcin Cre (Jackson Laboratory, Bar Harbor, ME, USA) and VEGF^fl/fl^ mice (Genentech, San Francisco, CA, USA). Tibiae were collected from either 4‐ or 16‐week‐old male and female mice and fixed in 4% paraformaldehyde (PFA). Skulls were frozen at –80  °C for µCT. To confirm conditional knockout of VEGF, genomic DNA was extracted from tail tips and genotyping performed as previously described.^5^ Use of animal tissue was carried out in compliance with Animals Act 1986.

### Micro‐computed tomography (µCT)

The Skyscan 1176 system (Bruker microCT, Kontich, Belgium) was used to scan the entire tibia and skulls; X‐ray tube potential of 45 kVp, X‐ray tube current of 556 µA, integration time of 375 ms, and voxel size of 18 µm. Slices were reconstructed, processed, and analyzed as previously described.^16^ A lower threshold of 80 was used to separate bone from non‐bone. Bone mineral density (BMD) was calculated by scanning a phantom of known hydroxyapatite content using the same conditions. The X‐ray attenuation coefficient was calculated in CTAn and used to calculate the BMD for each animal. CTvox (2.0) was used to determine bone threshold density within the tibia, by loading the bone‐density heat map function and using a standardized opacity for each bone. VGSTUDIO MAX (Volume Graphics, Heidelberg, Germany) was used to create 3D reconstructions of the skull scans. 2D images (Supplemental Fig. S1*A*, *B*) were imported into ImageJ for quantitative bone morphometry. For morphometric trabecular analysis, appearance of the trabecular bridge that connected the two primary spongiosa bone islands was set as a reference point for the analysis of proximal tibia metaphyseal trabecular bone; 10% of the total bone length from this point (towards diaphysis) was utilized.

For high‐resolution scans (0.65 µm), synchrotron‐based CT (SR CT) was conducted at the TOMCAT beamline of the Swiss Light Source (Villigen, Switzerland), following previously published methods.^17^ Porosity was extracted in ImageJ by thresholding two copies of the 300‐slice stack and inverting one copy. The “keep largest region” function was used and the stack eroded and dilated to create a mask. The image calculator plugin “AND” function was then used to combine the two images, leaving cortical porosity. Volumetric measurements of the porosity were extracted using “particle analysis” in ImageJ. Avizo (9.3.0; ThermoFisher Scientific, Waltham, MA, USA) was used for separation of osteocyte lacunae from vascular canals (open image file‐interactive thresholding‐label analysis‐sieve analysis‐volume rendering) and definition of threshold values. Individual absolute threshold values were used for each animal, based on the appearance of the first vascular canal. The threshold separating lacunae and canals was therefore set just below the volume of the smallest vascular canal (defined by shape and size; see Supplemental Methods). Analysis of the thickness, volume, and number for each fraction (osteocyte lacunae and vascular canals) was performed using the “Analyze Particles” function in ImageJ and defined using previously published nomenclature.^18^

Probability density distributions of lacunae volumes were estimated using the ggplot2^19^ package (version 2.2.1) in R (version 3.3.3),^20^ using a default bandwidth and 2048 estimate points. The volume corresponding to the threshold separating the two distinct populations of lacunae (low and high volume) was identified, allowing comparisons of the proportion of osteocyte lacunae in the smaller fraction between groups.

### Histochemistry

Contralateral left tibiae were prepared for histology as described previously.^21^ The Movat's Pentachrome staining protocol was used to enable identification of blood vessels.^22^ The % blood vessel area was calculated using the freehand selection tool on ImageJ (for area measurements).

### Scanning electron microscopy (SEM)

PMMA blocks created for histology were polished and uncoated surfaces imaged using a Zeiss EVO MA10 SEM operated at 20 kV and 49 Pa chamber pressure with a four‐quadrant backscattered electron (BSE) detector. Samples were treated to remove bone matrix and mineral by successive application of 2N HCl and 7% chlorine sodium hypochlorite solutions, removing calcified tissue and leaving a solid PMMA cast. To enable visualization of soft tissue, the surfaces of polished blocks and casts were stained using either ammonium triiodide or iodine vapor.^23^, ^24^

### Osteoblast cell culture

Long bone osteoblasts (LOBs) were isolated from 4‐day‐old (P4) male and female VEGF^fl/fl^ mice using 10 mg/mL collagenase and 4 mM EDTA, in a four‐step process.^25^

### Endothelial cell culture

Primary murine bone marrow endothelial cells were purchased from Generon (Slough, UK) and cultured according to manufacturer's guidelines.

### In vitro deletion of osteoblast‐derived VEGF

Once 80% confluent, LOBs were treated with adenovirus‐Cre‐GFP (Vector Laboratories, Burlingame, CA, USA) in growth media containing 10% FBS for deletion of VEGF or adenovirus‐GFP as a control at a multiplicity of infection of 100 for 6 days.^26^ For cell viability/proliferation only, LOBs were treated with Adenovirus‐Cre‐GFP (denoted OBVEGFKO) and Adenovirus‐GFP (denoted WT) for 24 hours. For collection of conditioned media (CM), cells were stepped down in low‐serum media (1%) for 24 hours.

### Cell viability/proliferation assay

Cells were plated at a density of 2500 cells per well and cultured for 24 hours for Cell Titer‐Glo 2.0 ATP assay (Promega, Madison, WI, USA) and BrdU Cell Proliferation ELISA (Abcam, Cambridge, UK).

### Alkaline phosphatase (ALP) analysis

Osteoblasts were plated at a density of 50,000 cells per well and left for 2 days to reach confluence. After incubation with adenovirus, they were fixed in ethanol and methanol: acetone for the ALP elution and staining, respectively, as described in the literature.^27^

### Automated cell counts

Cells were cultured on poly‐L‐lysine‐coated (50 µg/mL, 30,000 to 70,000 Mw) quartz coverslips (UQG Optics [Cambridge, UK] CFQ‐1017 #1.5, thickness 0.17 mm, Ø 10 mm) at a density of 10,000 cells, in preparation for Raman spectroscopy. After infection, cells were fixed with 4% paraformaldehyde and stained with Hoechst 33342 at a concentration of 100 ng/mL in the dark for 15 minutes. Images of fluorescent nuclei were acquired using the Deltavision Elite microscope at 10× magnification (GE Healthcare Life Sciences, Piscataway, NJ, USA) in combination with the SoftWoRx software and counted using ImageJ.

### Raman spectroscopy

Coverslips were prepared by sterilization in 100% ethanol before incubation in poly‐L‐lysine solution for 2 hours at 37  °C before being irradiated with UV light before plating. Cells were fixed in 4% PFA before spectral acquisition. Raman spectra were acquired using a Renishaw inVia Raman microscope equipped with a 532 nm laser and a Leica 63X water‐immersion microscope objective (NA of 1.2). Spectra were collected in the “Fingerprint region” from 600 cm^−1^ to 1750 cm^−1^. We have previously identified a range of protein bands associated with collagen and the extracellular matrix,^27^ which were also identified in this study and included CH_2_ deformation at 1450 cm^−1^, amide III, and amide I. Mineral bands were also detected, namely v_1_PO_4_^3‐^ (959cm^−1^), in addition to other weaker phosphate bands between 948 and 970 cm^−1^. Raman spectra were collected from the cytoplasm of 10 single cells, at five points around the distinctly visible nucleus with an exposure time of 20 seconds and three accumulations. Cosmic ray artefacts and background contribution from quartz were removed using WiRE 4.1. Preprocessing steps including wavelet denoising and background correction by fitting of a 9th‐order polynomial, were performed using iRootLab.^28^ Class means were normalized to the Raman peak at 1004 cm^−1^, corresponding to phenylalanine before deconvolution analysis using WiRE 4.1 as previously described.^27^

### qPCR and qPCR endothelial array

Total cell RNA was reverse transcribed, according to the manufacturer's instructions (Invitrogen, Carlsbad, CA, USA) and qPCR undertaken (Supplemental Table S1) using an annealing temperature of 60  °C. Ar, Esr1, and Esr2 primers were obtained from Biomol GmbH (Hamburg, Germany), and the remainder of sequences described were from Sigma (St. Louis, MO, USA). Expression levels were calculated using delta delta Ct values relative to GAPDH. The qPCR endothelial array described was from Qiagen (Valencia, CA, USA).

### Western blot analysis

LOBs and MBMEC were plated at density of 250,000 cells per 35 mm dish, protein lysed and used for Western blotting, as previously described.^29^ VEGFR2 and GAPDH rabbit monoclonal antisera were from Cell Signaling Technology (Danvers, MA, USA) and incubated 1:1000 in 3% BSA at 4  °C overnight.

### Immunohistochemistry

Tibiae were dissected and bones fixed in methanol‐free 4% PFA for 4 hours (4  °C). A 0.5 M EDTA solution was used for decalcification of the tibiae, followed by treatment with a solution of 20% sucrose, 2% polyvinylpyrrolidone in PBS at 4  °C. Tibiae were cryo‐embedded and 30 µm sections cut. Sections were permeabilized (0.3% Triton PBS) and blocked with 5% donkey serum, then incubated with sclerostin (SOST) primary antibody (R & D Systems, Minneapolis, MN, USA; 1:50) overnight (4  °C). Alexa Fluor 555 donkey anti‐goat (Invitrogen) was used as a secondary antibody (1:300). For CD31 primary (BD Biosciences, San Jose, CA, USA; 1:40) sections were incubated overnight (4  °C). Alexa Fluor 488 goat anti‐rat (Invitrogen) was used as secondary (1:400). Nuclei were stained with Hoechst 33342 (Invitrogen; 1:10,000) and sections mounted using Fluoromount G. Imaging was performed using a Zeiss Axioplan2 microscope.

### Statistics

For micro‐CT analysis in which direct comparisons between sex and genotype were made, two‐way ANOVA was employed. For WT versus OcnVEGFKO comparisons, one‐tailed paired *t* tests were undertaken between littermate animals, with WT representing VEGF^fl/fl^.

## Results

### Deletion of mature VEGF exerts sexually dimorphic effects on cortical bone microstructure

**Figure 1:**
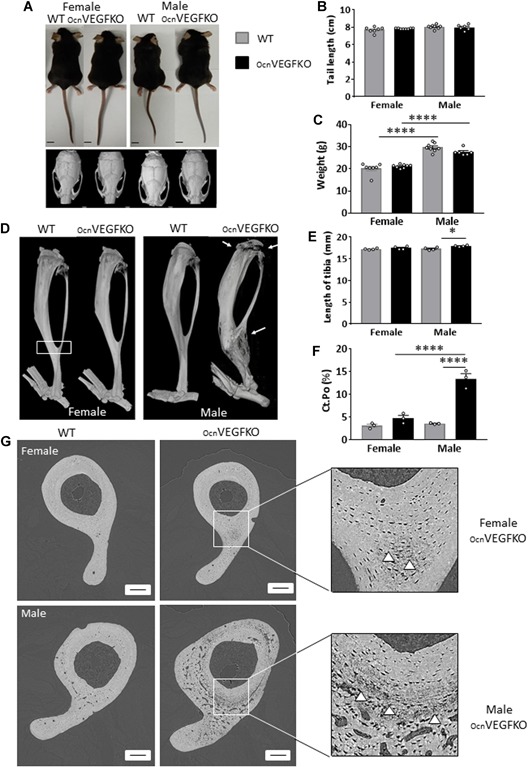
Conditional deletion of VEGF alters bone formation in adult tibiae. No gross anatomical differences were evident between OcnVEGFKO versus WT animals at 16 weeks of age (*A*; scale bar = 1 cm). Similarly, tail length (cm) was not significantly different between OcnVEGFKO and WT groups (*B*). No significant differences in the weight (g) of male OcnVEGFKO versus WT were observed (*C*) (*B, C, n* = 6–8 animals from 5 individual litters, presented as mean measurements ±SEM *p < 0.05, ****p < 0.0001 using two‐way ANOVA). Whole bone scans (*D*; 18 μm resolution) revealed poorly mineralized regions of the OcnVEGFKO tibia localized to the epiphysis and the tibiofibular junction (arrows) specifically in male animals. Tibial length (mm) was significantly greater in male OcnVEGFKO than WT (*E*; *n* = 4 males and 4 females from individual litters presented as mean ±SEM *p < 0.05 using two‐way ANOVA). Total volume porosity (*F*) was calculated after reconstruction of 300 SRCT slices lacunae (error bars indicate mean value ±SEM, *n* = 3 females and 3 males from individual litters ****p < 0.0001 using two‐way ANOVA). High‐resolution, synchrotron X‐ray computed tomography (SRCT) (*G*; 0.65 μm) slices from female and male WT and OcnVEGFKO mice revealed poorly mineralized areas of cortical bone at posterior region of tibiofibular junction (*G*; white arrows) and differences in cortical porosity between male and female OcnVEGFKO. Scale bar = 200 µm.

To study the skeletal functions of osteoblast‐produced VEGF, *Vegfa* was conditionally deleted in mature osteoblasts by crossing mice carrying floxed *Vegfa* alleles with mice expressing Cre recombinase under the control of the osteocalcin promoter.^30^, ^31^ No gross anatomical alterations were observed between 16‐week‐old *Vegfa* osteocalcin conditional knockout mice (OcnVEGFKO) of both sexes (Fig. 1 *A*, upper panel). Deletion of VEGF did not alter male and female tail length versus WT (Fig. 1 *B*). Although male WT and OcnVEGFKOs were observed to be significantly heavier than female WTs and OcnVEGFKOs, no differences were found between genotypes of either sex (Fig. 1 *C*). The skull phenotype was assessed using 10 cranial measurements made from medium‐resolution µCT scans (18 µm voxel size) in WT and OcnVEGFKO animals. Across the entire skull, there were no obvious defects in ossification when comparing male versus female and OcnVEGFKO versus WT mice (Fig. 1 *A*, lower panel). Craniometric measurements taken at the intramembranous neural crest, intramembranous mesoderm, and mandible were largely unaltered in OcnVEGFKO versus WT males and females. Changes were detected in the frontal length, which was significantly lower in the OcnVEGFKOs versus WT females, and the bitemporal distance was significantly lower in the OcnVEGFKO versus WT males (Supplemental Fig. S1, Supplemental Table S2).

Whole bone µCT scans were performed at medium resolution (18 μm voxel size) on tibiae from OcnVEGFKO and WT animals. Although no obvious differences were apparent in OcnVEGFKO versus WT females, severe and extensive porosity was evident particularly in male OcnVEGFKOs (Fig. 1 *D*, arrows; Supplemental Fig. S2 reveals fracture callus evident in one male OBVEGFKO animal; see video). At this resolution, the poorly mineralized bone phenotype was most evident in the epiphysis and the tibiofibular junction (Fig. 1 *D*). Tibial length was significantly increased in OcnVEGFKO versus WT male mice (Fig. 1 *E*).

The capabilities of standard lab‐based μCT can be significantly extended when synchrotron sources (SR) are used as the actual X‐ray source.^32^ High‐resolution (0.65 μm voxel size) SR CT of the tibiofibular junction (Fig. 1 *D*; box represents area scanned) revealed severe cortical porosity, particularly evident in the OcnVEGFKO male versus WT tibiae (Fig. 1 *F*, *G*). Intracortical porosity (Ct.Po, %) was not significantly increased in OcnVEGFKO females (Fig. 1 *F*); however, a large, significant increase was present in OcnVEGFKO males compared with WT. A significant increase in cortical porosity was also found in OcnVEGFKO males versus females.

### High‐resolution synchrotron X‐ray tomography revealed sexual divergence in vascular canal and lacunar phenotype after deletion of mature osteoblast‐derived VEGF in adult mice

**Figure 2:**
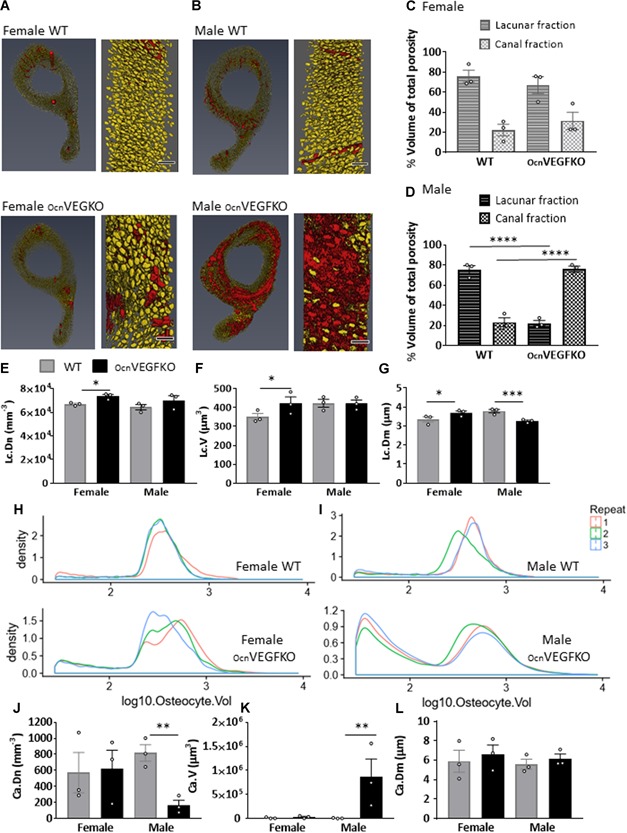
Distinction of bone porosity components is compromised after VEGF deletion in males. 3D renderings of osteocyte lacunae (yellow) and intracortical canals (red) from female (*A*) and male (*B*) WT and OcnVEGFKO mice (16 weeks old) after SRCT scans (0.65 μm voxel size). Scale bar = 50 µm. In WT male and female animals, osteocyte lacunae made up the highest fraction of the intracortical porosity (*C*). In male OcnVEGFKO, % pores constituting the intracortical canal fraction was increased versus WT (*D*). Measurements taken of regularly sized osteocyte lacunae show an increase in lacuna number density (Lc.Dn; *E*), mean lacunar volume (Lc.V; *F*), and mean lacunar diameter (Lc.Dm; *G*) in female OBVEGFKO versus WT. In males, there was a decrease in mean lacunar diameter (Lc.Dm; *G*) after knockout of OBVEGF. The probability density of osteocytes of different volumes for females (*H*) and males (*I*) are shown by graphs on a logarithmic scale, which were created using the free language and environment for statistical computing and graphics R (https://cran.r-project.org/). Each colored line represents one animal. Number of intracortical canals greater than the lacunar threshold (see Materials and Methods) is significantly decreased in male OcnVEGFKO (Ca.Dn; *J*). An increase in mean canal volume was observed after OcnVEGFKO (Ca.V; *K*); however, no change in mean canal diameter was observed in male OcnVEGFKO (Ca.Dm; *L*). Error bars indicate mean value ±SEM, *n* = 3 females and 3 males from individual litters *p < 0.05, **p < 0.01, ***p < 0.001, ****p < 0.0001 using two‐way ANOVA.

After SR CT, the intracortical canal network and osteocyte lacunae were extracted as a negative imprint of the calcified tissue from CT data sets.^17^, ^21^, ^33^, ^34^, ^35^, ^36^, ^37^ Using individual separation thresholds detailed (Supplemental Methods and Supplemental Table S3), 3D representations of the osteocyte lacunae (yellow) and intracortical canals (red) were generated as two different categories of the intracortical porosity (Fig. 2 *A*, *B*). Typically, in WT female animals, 75.8% of the total pore volume represented osteocyte lacunae and 22.0% intracortical canals (Fig. 2 *C*). In WT males, 75.4% of the pore volume consisted of osteocyte lacunae and 23.1% intracortical canals (Fig. 2 *D*). In OcnVEGFKO female animals, the ratio of lacunae versus canals was similar; however, in OcnVEGFKO males, a switch in the ratio of osteocyte lacunae to intracortical canals was identified, with the canal fraction accounting for 76.1% of the pore volume and lacunae only 22.3% (with the remainder categorized as noise).

Upon quantification of the separate intracortical fractions (Supplemental Table S3) OcnVEGFKO female mice displayed significantly increased lacunar number density (Lc.Dn; Fig. 2 *E*), mean lacunar volume (Lc.V; Fig. 2 *F*), and lacunar diameter (Lc.Dm; Fig. 2 *G*), compared with WT animals. In male OcnVEGFKO animals, there were no significant differences evident in the lacunar number density or mean lacunar volume, but a decrease in lacunar diameter was observed (Fig. 2 *G*).

To assess osteocyte size distribution between WT and OcnVEGFKO animals, we undertook a particle distribution analysis using the lacunar volumes. In WT female (Fig. 2 *H*) and male (Fig. 2 *I*) animals, the probability density curves of osteocyte lacunae size are dominated by a peak in the distribution above 200 μm^3^. In comparison, the OcnVEGFKO female animals (Fig. 2 *H*) displayed larger variation in lacunar volume between animals and a broader distribution curve. Male OcnVEGFKO animals (Fig. 2 *I*) presented a dramatically altered bimodal distribution, highlighting the presence of two distinct populations of osteocyte lacunae, one containing smaller lacunar volumes and one similar to WT volumes. The proportion of osteocyte lacunae in this smaller volume population was significantly greater in OcnVEGFKO males than in male WT (*p* = 0.007) and female OcnVEGFKO mice (*p* = 0.015). No significant difference in proportion was observed between OcnVEGFKO versus WT females (Supplemental Table S4 for raw data values).

OcnVEGFKO male animals displayed a reduced canal number density (Ca.Dn; Fig. 2 *J*) and an increased mean canal volume (Ca.V; Fig. 2 *K*); however, mean canal diameter (Ca.Dm; Fig. 2 *L*) was unchanged. When using animal‐specific volume thresholds (Supplemental Table S3 and Supplemental Fig. S3), where intracortical canals appear only at mean volumes greater than 7507 μm^3^ in OcnVEGFKO males (versus 1739 μm^3^ in WT animals), the current studies confirm there are significantly fewer but larger intracortical canals present in the tibiae of OcnVEGFKO male animals.

### High‐resolution synchrotron X‐ray tomography revealed sexual divergence in vascular canals after deletion of mature osteoblast‐derived VEGF in prepubertal mice

Androgen and estrogen bioactivity levels have been reported to increase between 5 and 7 weeks of age in male and female C57BL6 mice, respectively.^38^ The OcnVEGFKO cortical bone phenotype at 4 weeks of age (Supplemental Fig. S4*A–J*), where the levels of sex hormones will be similar,^38^ was also examined. Some sexual dimorphism was observed, specifically in Ca.V in male OcnVEGFKO versus females at 4 weeks of age (Supplemental Fig. S4*I*). In addition, significant increases in the canal fraction (% volume of total porosity) were observed in male OcnVEGFKO versus WT and reduction in lacunae fraction (% volume of total porosity) in male OcnVEGFKO versus WT. The 4‐week‐old morphological cortical synchrotron CT data are summarized along with 16‐week‐old data (Supplemental Table S5).

### Effect of mature osteoblast‐derived VEGF deletion on vascular and osteocyte phenotype

**Figure 3:**
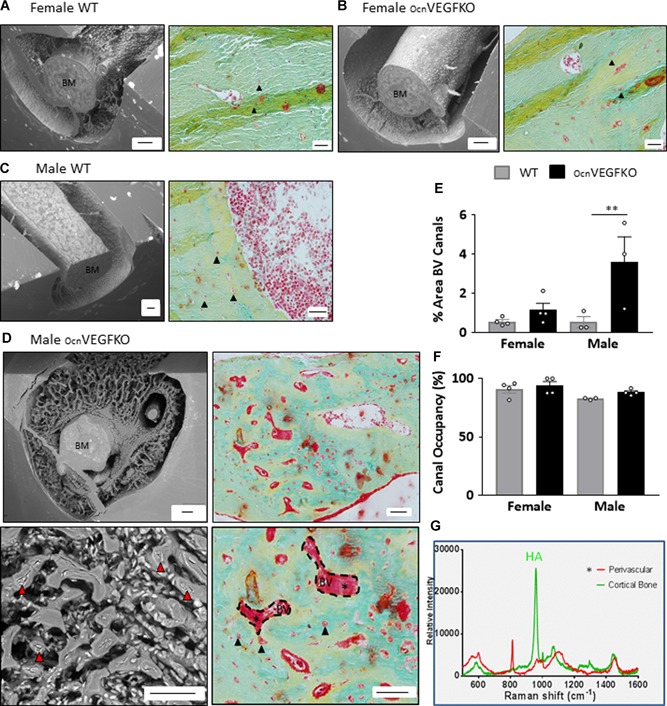
Conditional VEGF deletion increases blood vessel area and unmineralized osteoid in males. No obvious differences in bone architecture are found between female WT (*A*) and OcnVEGFKO bones (*B*) in either the SEM images (left; scale bar = 100 µm) or the pentachrome‐stained bone sections (right; scale bar = 20 µm). SEM shows very few blood vessels remaining in both the WT and OcnVEGFKO after treatment with HCl and NaOCL. There are minimal areas of osteoid surrounding the BV canals and osteocyte lacunae. In comparison to male WT (*C*), in OcnVEGFKO bones (*D*), increased vascularization (red arrows) was present and visible in SEM images after treatment with HCl and NaOCL (left) and in pentachrome‐stained images (right). The close proximity and overlapping of the osteocytes surrounding the bone marrow (BM) and the poor mineralization result in large amounts of PMMA remaining. In histologically stained male OcnVEGFKO sections, there were large amounts of unmineralized osteoid (*) surrounding BV canals (hatched lines). Analysis of stained sections showed that OcnVEGFKO versus WT % area BV canals (*E*) was increased in males. There was a high level of vascular canal occupancy (*F*), which remained unchanged in OcnVEGFKO versus WT animals (error bars indicate mean value ±SEM, *n* = 4 female and *n* = 3 male mice from individual litters, **p < 0.01 using two‐way ANOVA). Raman spectroscopy revealed a lack of hydroxyapatite (HA) in perivascular regions versus cortical bone (*G*).

BSE‐SEM imaging was used to enable identification of the cellular components of the bone cortex at the tibiofibular junction, and after erosion of the bone mineral with HCl and bleach (NaOCL), the cortical vasculature was revealed (Fig. 3 *A–D*). No differences were apparent between WT (Fig. 3 *A*) and OcnVEGFKO females (Fig. 3 *B*) after HCl and NaOCL treatment. In contrast to females and consistent with the porous phenotype observed in OcnVEGFKO mice after SR CT imaging, we observed vascular canals densely populating the bone cortex in OcnVEGFKO males in marked contrast to WT animals (Fig. 3 *C*). Lack of mineral directly surrounding the vasculature was evident in OcnVEGFKO males (Fig. 3 *D*), and after HCl/NaOCL treatment, large areas of PMMA were retained because of the extensive overlapping of osteocytes covering the blood vessels and poor mineralization. This phenotype highlighted a lack of matrix organization within the cortical bone of OcnVEGFKO males, closely resembling woven bone, which is characteristically laid down rapidly and provides a structural scaffold for processes such as fracture healing.

BSE SEM imaging of PMMA‐embedded tibiae stained with ammonium triiodide enabled examination of the effect of VEGF deletion on matrix mineralization longitudinally. As observed in the cross‐sectional images, in OcnVEGFKO female tibia sections, the bone cortex appeared comparable to WT animals (Supplemental Fig. S5*A*). In contrast, in OcnVEGFKO male bones, the bone was not mineralized efficiently and extensive areas of osteoid in the cortex surrounding the blood vessels and osteocytes (Supplemental Fig. S5*B*).

The poorly mineralized and highly vascularized phenotype present in the tibia of OcnVEGFKO males was further verified by histology (Fig. 3 *A–D*), using Pentachrome staining to distinguish between nonmineralized (red) and mineralized components (green) of the bone. A significant increase was highlighted in the area of vascular canals in OcnVEGFKO males versus WT animals (Fig. 3 *E*). Vascular canal occupancy was also assessed, but no significant differences were identified (Fig. 3 *F*). Similar to the BSE imaging of OcnVEGFKO males after HCL/NaOH treatment, Pentachrome staining revealed large areas of unmineralized osteoid surrounding blood vessel canals and osteocyte lacunae (Fig. 3 *D*), which contributed to the elevated pore volume detected by SR CT. To identify matrix components of the perivascular and lacunar osteoid detected by the Pentachrome stain, Raman spectroscopy was utilized (Fig. 3 *G*) and confirmed that this region was completely devoid of hydroxyapatite in contrast to the mineralized bone matrix (stained in green). No difference in endosteal remodeling was observed between OcnVEGFKO versus WT (Supplemental Fig. S6).

### Effects of osteoblast‐derived VEGF deletion on trabecular bone

Given the extent of the mineralization defect observed in the cortical bone after VEGF deletion, we also analyzed trabecular bone phenotype (Supplemental Fig. S7*A–E*). No sexual dimorphism was observed in any trabecular bone parameters in OcnVEGFKO animals versus WT at 4 weeks (BV/TV, % bone volume; BS/TV, bone surface area; Tb.Th, trabecular thickness; Tb.N, trabecular number). At 16 weeks, a significant reduction in trabecular number (Tb.N (1/U)) was observed in female OcnVEGFKO versus WT (Supplemental Fig. S7*E*).

### In vitro VEGF deletion in osteoblasts does not alter endothelial cell viability or proliferation

To investigate the role that sex hormones may play in the sexual divergence of the in vivo phenotype observed, we undertook in vitro experiments to remove the effects of circulating sex hormones. The experimental approach centered around deletion of VEGF from osteoblasts (using adenoviral cre recombinase; OBVEGFKO) isolated from the long bones of postnatal day 4 (P4) male or female VEGF^fl/fl^ animals (WT). We subsequently investigated indirect paracrine effects on bone‐marrow endothelial cell behavior.

**Figure 4:**
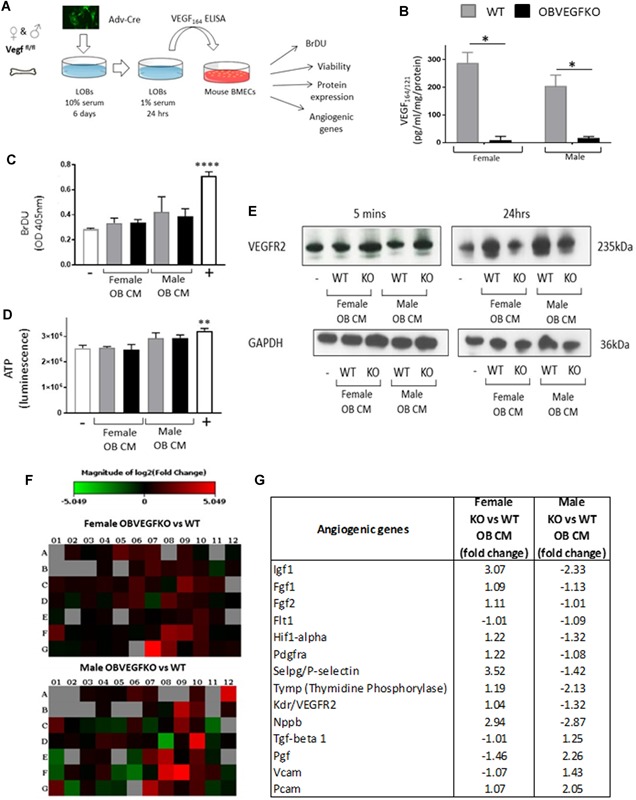
Sexually dimorphic effects of VEGF deletion on endothelial cell function. Mouse bone marrow endothelial cells (MBMECs) were treated with LOB conditioned media (CM) from male and female WT and OBVEGFKO cells for 24 hours (*A*). VEGF ELISA confirms VEGF deletion (*B*; three separate experiments ±SEM, *p < 0.05 using *t* test). For proliferation (BrDU; *C*) and viability (ATP; *D*) assays, MBMECs were treated for 24 hours with WT or OBVEGFKO CM with low‐serum (1%) media (–) and high serum (10%; +) used as controls. Data represent *n* = 6 replicates ±SEM, **p < 0.01, ****p < 0.0001 using *t* test. For VEGFR2 expression, MBMECs were treated for either 5 minutes or 24 hours and total VEGFR2 protein assessed by Western blotting (*E*). Gene expression changes were assessed by endothelial qPCR array with heat maps representing expression changes (fold change) of 84 different genes after the treatment of MBMEC with male and female OBVEGFKO and WT conditioned media (*F*). Table summarizes changes in gene expression in both sexes (*G*).

Endothelial cells express high levels of VEGFR2 protein in vitro,^29^ and a paracrine communication has been hypothesized between the vascular endothelium and OBs and indirect effect of VEGF on osteoblast via endothelial cells reported.^29^ Herein, we sought to establish whether any sexually dimorphic mechanisms regulating the OBVEGFKO bone phenotype are driven indirectly by vascular endothelial cells. In vitro conditioned media (CM) experiments utilizing male and female LOBs with VEGF‐deleted and murine bone marrow endothelial cells (BMECs) were used to examine this mechanism (Fig. 4 *A*). Although VEGF deletion was >90% in both female and male OBVEGFKO cultures (Fig. 4 *B*), OBVEGFKO CM did not alter endothelial cell proliferation (Fig. 4 *C*) or cell viability (Fig. 4 *D*) versus WT. Surprisingly, MBMECs treated with male and female OBVEGFKO CM for 5 minutes displayed enhanced levels of VEGFR2 protein versus WT (Fig. 4 *E*), which may be reflective of higher VEGF levels present in WT LOBs (Fig. 4 *B*), potentially inducing internalization of the VEGFR2. In contrast, after 24 hours of exposure of male and female OBVEGFKO CM, VEGFR2 protein levels in MBMECs were reduced versus WT (Fig. 4 *E*), suggesting that long‐term deletion of OBVEGF could impact endothelial cell sensitivity to VEGF. These results suggest that the sexual dimorphic effect observed in the vasculature in vivo, after OBVEGFKO is either 1) not linked to altered endothelial cell viability, proliferation, or VEGFR2 expression and/or that 2) sex steroids and mechanical or environmental factors are required to induce the vascular effects observed in vivo. To investigate this further, we undertook an “endothelial” qPCR array to assess alterations in BMEC endothelial cell gene expression. After 24 hours of exposure to male and female OBVEGFKO CM, widespread sexually divergent alterations in BMEC gene expression versus WT CM were observed (Fig. 4 *F*). Angiogenic growth factors such as transforming growth factor‐β1 and placental growth factor mRNA were both upregulated (+1.5‐fold and +2.26‐fold, respectively) in BMEC treated with male OBVEGFKO conditioned media versus WT but reduced after treatment with female OBVEGFKO media (–1.01 and –1.46, respectively). In addition, mRNA expression levels were upregulated for a number of adhesion molecules including VCAM (+1.43) and PECAM (+2.03) in BMECs treated with male OBVEGFKO CM versus WT, which may contribute to the increased vasculature observed in vivo after VEGF deletion. Other genes that may affect vascular function which were downregulated in ECs treated with male OBVEGFKO media include P‐selectin (–1.42) and thymidine phosphorylase (tymp), FGF1 (–1.13), and FGF2 (–1.01). Sex steroids and the growth hormone–insulin‐like growth factor 1 (IGF1) axis have been shown to interact closely during puberty,^39^ and IGF‐1 has previously been reported to be a primary determinant of the development of the skeletal dimorphism. Interestingly, we have found that IGF‐1 is upregulated in BMECs treated with female OBVEGFKO media (+3.07) and which is conversely downregulated in BMECs treated with male OBVEGFKO media (–2.33) and thus potentially contributing to the skeletal phenotype observed (Fig. 4 *G*).

### In vitro VEGF deletion induces distinct matrix and mineralization signatures in male and female osteoblasts

To investigate whether the observed defect in mineralization in male OBVEGFKO mice was driven by direct effects of VEGF on LOB function, we deleted VEGF with adenovirus‐Cre‐GFP as previously described.^26^ Consistent with our previous work,^29^ LOBs did not express detectable levels of VEGFR2 protein or mRNA (Supplemental Fig. S8*A*, *C*). This was similar to the information derived from immunohistochemical labeling of bone sections for VEGFR2, which co‐localized only with CD31 endothelial cells (Supplemental Fig. S8*B*) and was further validated by qPCR (Supplemental Fig. S8*C*).

**Figure 5:**
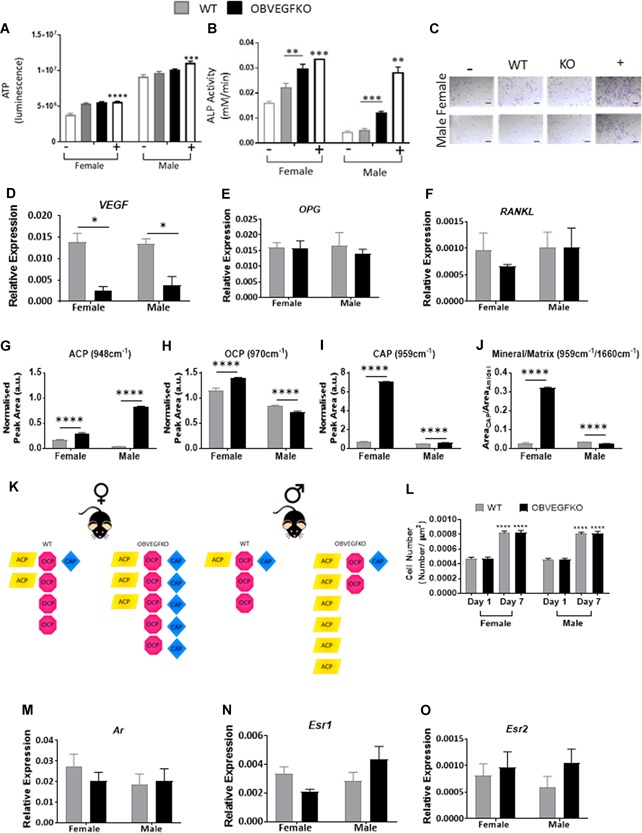
Direct effects of VEGF deletion on male and female osteoblast function. VEGF was deleted in vitro in male and female LOBs (P4). No differences in viability (ATP; *A*) versus WT were evident from males or females. Control cells received low‐serum media (–) Wnt3a (75 ng/mL +). Data represent mean value from six individual infections ±SEM, ***p < 0.001, ****p < 0.0001 using *t* test. Significant increases were found in alkaline phosphatase (*B*) after OBVEGFKO (*C*; scale bar = 100 µm). Control cells received 10% FBS media (–) and 10% FBS containing β‐glycerol‐phosphate (+). Data represent mean value from three individual infections ±SEM, **p < 0.01, ***p < 0.001 using *t* test. Knock‐down efficiency was further confirmed using qPCR (*D*) for VEGF mRNA in male and female OBVEGFKOs. The involvement of osteoclasts in the phenotype was investigated looking at relative expression of OPG (*E*) and RANKL (*F*), where no significant changes were identified after OBVEGFKO. Raman spectroscopy was able to detect clear and significant sex differences (between immature (ACP; *G*), intermediate (OCP; *H*), and mature (CAP; *I*) calcium species in WT and OBVEGFKO cells. Mineral/matrix ratio also shows a significant increase in matrix maturity after OBVEGFKO in females, whereas the converse is exhibited in males (*J*). Schematic representation of the changes in calcium phosphate species in WT and OBVEGFKO cells are shown (*K*). Error bars indicate mean value ±SEM, **p < 0.01, ****p < 0.0001 using *t* test, *n* = 50 spectra from each treatment group. Cell number increased over time in culture (****p < 0.0001) in both sexes but was not affected by OBVEGFKO (*L*). Data represent mean number of nuclei per µm^2^ ±SEM, ****p < 0.0001 using two‐way ANOVA, *n* = 20 fields of view per group. No notable changes in gene expression of androgen receptor (Ar; *M*), estrogen receptor 1 (Esr1; *N*), or estrogen receptor 2 (Esr2; *O*) were evident in males and females after OBVEGFKO.

Deletion of VEGF did not impact male or female LOB viability (Fig. 5 *A*). However, in contrast, deletion of VEGF in both male and female LOBs significantly increased ALP activity (Fig. 5 *B*, *C*), suggestive of autocrine/intracrine regulation given the lack of VEGFR2 expression. Reduced VEGF mRNA expression was confirmed by qPCR after adenovirus‐Cre‐GFP treatment (Fig. 5 *D*). The potential communication of male and female OBVEGFKO cells with osteoclasts was investigated by measurement of OPG and RANKL mRNA levels, but no differences were observed versus WT (Fig. 5 *E*, *F*).

We have previously identified Raman spectroscopy as a highly sensitive fingerprinting technique for specific detection of differentiation changes in primary bone cell cultures.^27^ Given its enhanced sensitivity, we utilized Raman spectroscopy to identify in detail any alterations in mineral and matrix components in LOBs after VEGF deletion (Supplemental Fig. S9*A*). Raman spectra (Supplemental Fig. S9*B*) were collected within the “fingerprint region” and subsequent spectral peak analysis and assignment was carried out on mean spectra. Upon analysis of female LOBs with VEGF successfully deleted (Supplemental Fig. S9*C*, *D*), small but significant changes in amorphous calcium phosphate (ACP) (Fig. 5 *G*) and octacalcium phosphate (OCP; Fig. 5 *H*) (+1.77‐fold and +1.21‐fold, respectively) were observed; however, the largest fold change in mineral species was found in carbonated apatite (CAP; Fig. 5 *I*; +10.5‐fold). The mineral‐to‐matrix ratio in OBVEGFKO female cells was also increased (Fig. 5 *J*; +11.9‐fold), suggesting that when VEGF is deleted, females exhibit a mature mineral signature (Fig. 5 *J*). Male OBVEGFKO cells had their largest fold change evident in the levels of immature ACP (Fig. 5 *G*, *K*; +23.6‐fold), consistent with a small but significant decrease in mineral‐to‐matrix ratio (Fig. 5 *J*; –1.33‐fold). A reduction in OCP was also evident in male OBVEGFKO cells (–1.17‐fold) with a small significant increase in CAP (+1.24‐fold). To control for cell number, male and female WT and OBVEGFKO cells were counted at day 1 and day 7 after plating on quartz coverslips. No significant differences in cell number were observed between sex or genotype at each time point (Fig. 5 *L*). To investigate whether steroid signaling responses were involved in driving this sexually dimorphic effect, we measured androgen receptor (Ar) and estrogen receptor 1/2 mRNA expression (Fig. 5 *M–O*) in male and female WT and OBVEGFKO cells. No significant differences were identified across sex or genotype.

### Deletion of osteoblast‐derived VEGF increases sclerostin expression in male bones

**Figure 6:**
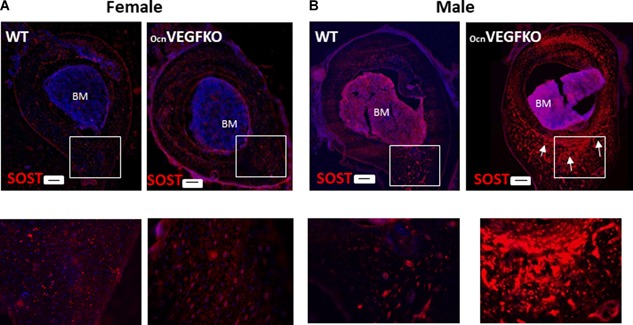
Sexual dimorphic alterations in protein expression of SOST after VEGF deletion are evident in whole bone sections. Cryosections from female (*A*) and male (*B*) WT and OcnVEGFKO tibiofibular junction were stained with sclerostin primary antibody and Hoechst to stain the nuclei. Increases in sclerostin levels were visible after OcnVEGFKO in males (Alexa Fluor 555), specifically in the posterior region (arrows) below the bone marrow (BM). White box represents the region of cortical bone magnified below. Scale bar = 100 µm.

Given the mineralization defect described between male and female OcnVEGFKO animals and cells, we investigated whether expression levels of an established inhibitor of mineralization, sclerostin (SOST), was influenced. Consistent with our CT and histology analyses, cryo sections were cut at the tibiofibular junction of WT and OcnVEGFKO male and female mice and labeled with sclerostin antisera. In WT and OcnVEGFKO female mice, sclerostin staining was observed to be low and localized to osteocyte populations (Fig. 6 *A*). In WT males, the levels of sclerostin expression appeared highest at the tibiofibular junction. In contrast, in OcnVEGFKO animals, sclerostin expression was high and widespread across cells within the entire bone section (Fig. 6 *B*).

### Adult males conserve parameters of tibial geometry and shape after OB VEGF deletion

**Figure 7:**
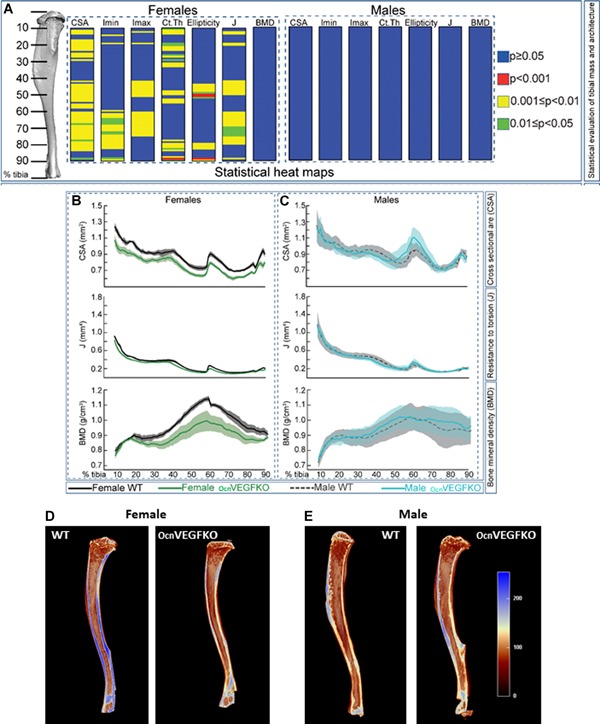
Significant alterations in tibial geometry after bone‐derived VEGF deletion is evident only in females. Minimum and maximum second moments of inertia (I_min_ and I_max_, respectively), cross‐sectional area, resistance to torsion (J), ellipticity, cortical thickness, and bone mineral density (BMD) of male and female OcnVEGFKO tibiae versus WT at 16 weeks of age (*A*). Graphical heat map summarizes statistical differences (using ANOVA) at specific matched locations along the tibial length (10% to 90%), representative of overall effect of genotype. Red *p* ≤ 0.0001, yellow *p* ≤ 0.001–0.01, green *p* ≤ 0.01–0.05, and blue *p* ≥ 0.05. Line graphs represent means for female (*B*) and male (*C*) WT versus OcnVEGFKO ±SEM (*n* = 4 female and 4 male mice from individual litters). Longitudinal tibial cross sections created using CTvox show a decrease in bone density in OcnVEGFKO females (*D*) and an increase in bone density in OcnVEGFKO males (*E*). Heat map scale plotted from 0 (brown; low‐threshold density) to 255 (blue; high‐threshold density).

After our observed alteration in matrix mineralization, we finally sought to analyze the impact of OBVEGF deletion on whole bone geometry and shape of male and female bones. A graphical heat map representation of statistical significance of the effect of VEGF deficiency on measures of bone mass and shape (Fig. 7 *A*), including measurements of cross‐sectional area (CSA), minimum moment of inertia (I_min_), maximum moment of inertia (I_max_), and cortical thickness (Ct.Th), ellipticity (bone shape) and predicted resistance to torsion or polar moment of inertia (J) identified significant differences at different points along the tibial length in females only. Measures of bone mass (CSA) and shape (J) are significantly compromised in female (green; Fig. 7 *B*), but not male (turquoise; Fig. 7 *C*) OcnVEGFKO mice compared with WT controls (grey). It appears that while males compromise on bone porosity after OcnVEGFKO, tibial geometry and mechanical properties of the bones are maintained. BMD measurements along the entire tibia were not significantly different between male and female OcnVEGFKO versus WT bones (Fig. 7 *A–C*).

Correlating with these changes in bone geometry found predominantly in females, analysis of longitudinal tibial cross sections highlighted a decrease in bone threshold density in females after OcnVEGFKO (Fig. 7 *D*). In contrast, male tibiae appear to compensate for the lack of VEGF, and an increase in bone threshold density was observed after OcnVEGFKO (Fig. 7 *E*).

Male versus female comparisons of bone geometry showed basal differences in cross‐sectional cortical thickness in WT animals (Supplemental Fig. S10*A*), with female bones significantly thicker than in males at several points above and below the tibiofibular junction. BMD measurements were not significantly altered when comparing female versus male tibiae (Supplemental Fig. S10*A–C*). After OcnVEGFKO, the sex differences in thickness at these specific regions were no longer significant. In marked contrast to WT bones (Supplemental Fig. S10*B*), OcnVEGFKO bone measurements (Supplemental Fig. S10*C*) of CSA, I_min_, I_max_, Ct.Th, and J were significantly greater in males versus females at several points along the tibial length. The only exception to this is ellipticity, which was significantly higher in female versus male OcnVEGFKO animals at ~80% along the tibial length (Supplemental Fig. S10*A*).

As sexual dimorphism in vascular canal parameters were evident by SRCT in 4‐week‐old male OcnVEGFKO mice. Whole bone (tibial) geometry was also assessed in 4‐week‐old animals (Supplemental Fig. S11*A*) and included CSA, I_min_, I_max_, Ct.Th, ellipticity, and J. Significant differences in CSA, I_max_, Ct.Th, and ellipticity (Supplemental Fig. S11*B*) were identified in multiple sites of male OcnVEGFKO versus WT.

## Discussion

**Figure 8:**
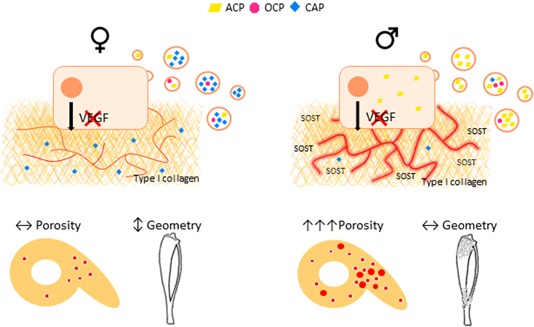
Sexually dimorphic effects of VEGF deletion on matrix mineralization and porosity link to whole bone phenotypes. Female OcnVEGFKO mice are able to maintain porosity and vasculature but compromise tibial geometry after deletion of VEGF in osteoblast cells. In contrast, male OcnVEGFKOs are able to maintain tibial geometry, despite increased cortical porosity associated with altered blood vessel configuration. Raman spectroscopy has shown that this skeletal sexual dimorphism could be linked to direct effects of VEGF on OB matrix production, with increased immature phosphate species (ACP) predominant in male OBVEGFKO cells.

The current studies demonstrate that VEGF produced by osteocalcin‐positive cells exerts sexually dimorphic effects on bone mineralization and vascularization, driven in part by direct effects on OB differentiation potential (summarized in Fig. 8). Using a range of approaches to assess the differences in cortical bone architecture, we observed enhanced cortical porosity in OcnVEGFKO males compared with females. Dimorphism in porosity was demonstrated further with fewer, larger intracortical canals and less osteocyte lacunae present in male OcnVEGFKOs. The increase in canal volume in male OcnVEGFKOs was associated with the appearance of two distinct populations of osteocyte lacunae, one containing smaller lacunae and one of similar size to WTs. Extensive unmineralized osteoid resembling immature woven bone was evident encircling the intracortical blood supply and osteocyte lacunae in male OcnVEGFKOs. We investigated this vascular defect further in vitro, and a paracrine role for OB‐derived VEGF was evidenced via the sexually dimorphic alteration in pro‐angiogenic gene expression of bone marrow–derived endothelial cells after VEGF deletion. Direct effects of OB‐derived VEGF on OB matrix were also evident in vitro in male OBVEGFKO cells producing predominantly immature mineral precursors (amorphous calcium phosphate) versus mature mineral (carbonated apatite) in female OBVEGFKO cells. The matrix alterations were independent of circulating hormones and androgen and estrogen receptor mRNA expression levels and suggest an underlying genetic influence. The failure for OBs to mineralize efficiently was associated with enhanced SOST levels in male OcnVEGFKO bones. Finally, our studies of whole bone geometry revealed male OcnVEGFKOs maintained bone shape despite increases in cortical porosity.

Our findings describe the concept that sexual dimorphism in VEGF signaling was linked to production of matrix components in bone controlled partly indirectly by alterations in endothelial cell gene expression and also directly by osteoblast matrix production. We have described how VEGF‐dependent sexual dimorphism appears to underlie differences in phosphate repertoires and unmineralized matrix components produced by male and female‐derived osteoblasts in vitro, which may directly recapitulate in the mineralization defect described in vivo. Endogenous VEGF has been shown to be critical in the regulation of mineralization,^40^, ^41^, ^42^, ^43^ although VEGF effects have never previously been addressed in the context of sexual dimorphism. Our results show that bone‐derived VEGF can differentially control rates of mineralization between male and females, which could potentially support the different bone growth rates of both sexes and underlie bone matrix composition in later life.

Vascular endothelial cells express high levels of VEGF receptor 2, and a paracrine role for OB‐derived VEGF has been proposed^3^ with endothelial cells known to produce osteogenic factors and influence bone formation.^44^, ^45^ When VEGF was deleted in male and female OB cultures, endothelial cell viability and proliferation was maintained, suggesting that osteoblasts may produce other angiogenic factors to compensate for the lack of VEGF, in order to support endothelial cell survival. Furthermore, endothelial cells treated with male or female OBVEGFKO conditioned media altered their gene expression in a sex‐specific manner, which included increased IGF‐1 in females and decreased IGF‐1 levels in males. Aside from growth factor production, it is also possible that the sexually dimorphic vascular phenotype observed in vivo could be due to alterations in the osteoblast matrix constituents impacting angiogenesis directly. The extracellular matrix (ECM) is known to control vascular ingrowth with the temporal and spatial regulation of ECM remodeling providing a scaffold to control cell growth, migration, and differentiation during different stages of angiogenesis. ECM molecules are also emerging as key effectors of SOST inhibition as they are thought to regulate the availability of sclerostin to its osteoblastic targets.^46^ The failure of OcnVEGFKO male bones to mineralize effectively is consistent with this idea as we have identified increased SOST protein expression in the bones of these mice. Furthermore, in line with our observations that SOST effects appear to be sexually dimorphic, Mödder and colleagues^47^ reported a correlation between circulating SOST levels with age, sex, and bone mass in a large population‐based study with men having higher serum SOST levels than women with age.

Today the evidence supporting a role of VEGF in osteoporosis exists and its requirement for fracture repair is well documented. Bone shape, size, and density are accepted clinical predictors of fracture susceptibility and single measurements of bone mass are now thought to be ineffective in predicting bone fragility. The effects of VEGF deficiency on measures of bone mass and shape identified most significant differences along the tibial length at 16 weeks only in female OcnVEGFKO versus WT. Female OcnVEGFKO bones were thinner, with a smaller cross‐sectional area and less resistance to bending and torsional loading. In contrast, significant differences were observed in OcnVEGFKO versus WT males at 4 weeks of age and included cortical thickness and ellipticity, which were no longer present by 16 weeks. This indicates that after the loss of bone‐derived VEGF, males may override early alterations in bone shape at the expense of cortical porosity into adulthood. Further, this implies that as adults, male OcnVEGFKOs may either attempt to overcome the mechanical instability associated with increased porosity by maintaining bone shape or that bone porosity and bone shape are two independently regulated mechanisms.

Sex differences in the skeleton are present in puberty, adulthood, and old age, and a wealth of evidence has demonstrated sex steroids as key regulators of bone health and mechanical responsiveness throughout life. Our results are relevant as they suggest systemic loss of VEGF, eg, with age could impact bone health differently in men and women. Given the current demonstration of sexual dimorphism in cortical porosity and mineralization driven by VEGF in vivo and in vitro at the cellular level, future studies should aim to isolate the contribution of sex steroids completely by hormone depletion in vivo after VEGF deletion. Such studies could reveal underlying sex‐specific genetic regulators of bone mineralization, which could be targeted therapeutically. This is particularly important in an aging demographic given the failure of hormone replacement therapy to treat severe cases of osteoporosis. Furthermore, these findings are extremely timely in the context of increased prevalence of degenerative bone disease in males and critically given reports of divergent sex effects on post‐fracture mortality.^48^, ^49^

## Disclosures

All authors state that they have no conflicts of interest.

## Supporting information

Additional Supporting Information may be found in the online version of this article.

Supporting information.

Supporting information.
