# Supplementary material for: Regulation of the Bone Vascular Network is Sexually Dimorphic
Source: J Bone Miner Res. 2019 Oct 9;34(11):2117–32. doi: 10.1002/jbmr.3825 (PMC6899569; doi:10.1002/jbmr.3825)
Supplement: Supplementary file 2 — Supporting information. [file JBMR-34-2117-s002.pdf]

Supplementary table S1: Forward and reverse sequences for primers.

Gene of interest	Forward sequence	Reverse sequence
VEGF	ATCTTCAAGCCGTCCTGTGT	CTGCATGGTGATGTTGCTCT
OPG	AGTCCGTGAAGCAGGAGT	CCATCTGGACATTTTTTGCAAA
RANKL	CACAGCGCTTCTCAGGAGCT	CATCCAACCATGAGCCTTCC
Ar	GGACCATGTTTTACCCATCG	TCGTTTCTGCTGGCACATAG
Esr1	TTCTCCCTTTGCTACGTCAC	ATCGCTTTGTCAACGACTTC
Esr2	TGGTCATCAAATCGACCTTT	GGAACAAGGTCACATCCAAG
VEGFR2	TCTGTGGTTCTGCGTGGAGA	GTATCATTTCCAACCACCCT
GAPDH	TGTGTCCGTCGTGGATCTGA	CCTGCTTCACCACCTTCTTGA

Supplementary figure S1

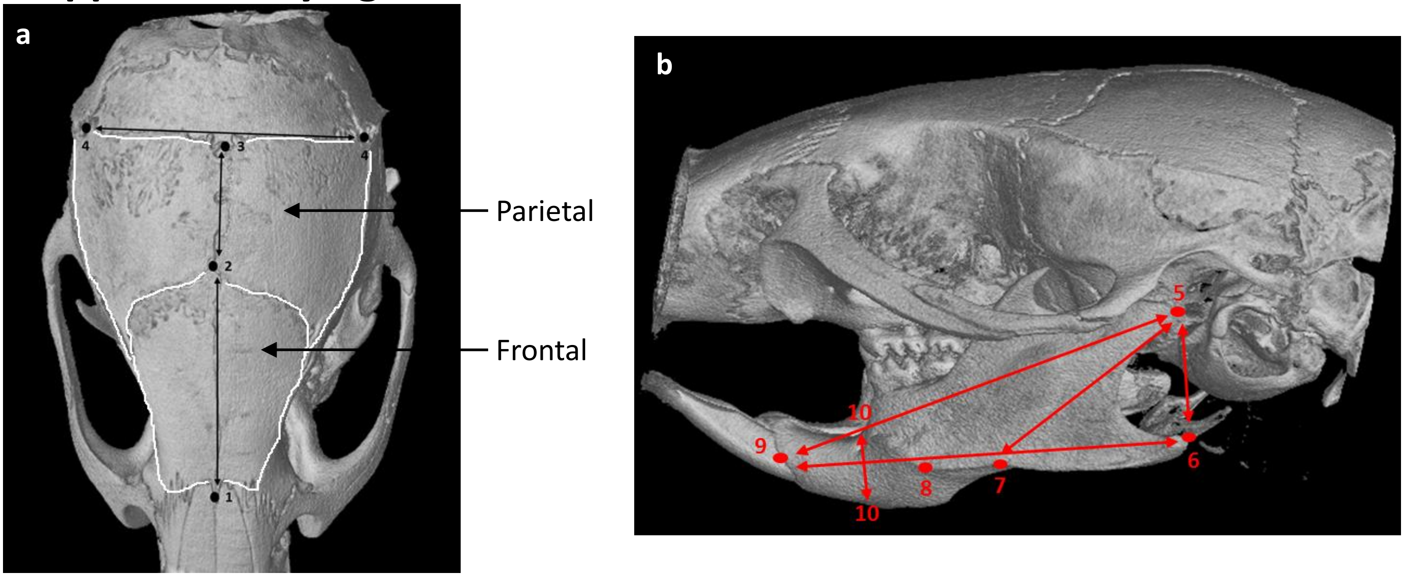

Supplementary figure S1. Effects of sex on skull phenotype following VEGF deletion. Craniometric measurements of 16 week old male and female WT and OcnVEGFKO were taken from reconstructed Skyscan1172 micro-CT images (18µm). The schematic (a, b) shows the landmarks used to take cranial measurements; 1–2: frontal length, 2–3: parietal length, 4-4: bitemporal distance, 5-6: posterior mandible height, 5-7: condylar axis, 5-9: effective mandible length, 6-9: mandible plain, 7-8: mandible axis, 10-10: anterior mandible height[50].

Supplementary table S2: Craniometric measurements for 16 week old male and female WT and OcnVEGFKO animals.

Craniometric measurements	Female WT	Female OcnVEGFKO	P value	Male WT	Male OcnVEGFKO	P value
	**n=4**	**n=4**	**Female WT vs OcnVEGFKO**	**n=3**	**n=4**	**Male WT vs OcnVEGFKO**
**Intramembranous (neural crest)**						
Frontal length (mm)	7.97 ± 0.16	7.41 ± 0.081	< 0.01	8.093 ± 0.045	7.79 ± 0.086	NS
Frontal area (mm^2)	33.52 ± 0.84	33.27 ± 0.82	NS	35.40 ± 0.30	33.97 ± 2.028	NS
	**n=4**	**n=4**	**Female WT vs OcnVEGFKO**	**n=4**	**n=4**	**Male WT vs OcnVEGFKO**
**Intramembranous (mesoderm)**						
Bitemporal distance (mm)	9.79 ± 0.038	9.67 ± 0.053	NS	9.84 ± 0.043	9.46 ± 0.084	< 0.01
Parietal length (mm)	4.29 ± 0.15	3.92 ± 0.12	NS	4.40 ± 0.081	4.24 ± 0.13	NS
Parietal area (mm^2)	54.017 ± 0.46	52.94 ± 0.75	NS	53.78 ± 1.075	54.73 ± 1.097	NS
	**n=4**	**n=4**	**Female WT vs OcnVEGFKO**	**n=4**	**n=4**	**Male WT vs OcnVEGFKO**
**Mandible, intramembranous (neural crest)**						
Effective mandible length (mm)	11.98 ± 0.24	11.72 ± 0.56	NS	13.65 ± 1.20	11.39 ± 0.31	NS
Mandible axis (mm)	2.49 ± 0.15	2.57 ± 0.21	NS	2.72 ± 0.26	2.18 ± 0.099	NS
Condylar axis (mm)	6.64 ± 0.20	6.53 ± 0.17	NS	7.63 ± 0.60	6.51 ± 0.21	NS
Anterior mandible height (mm)	1.92 ± 0.067	2.35 ± 0.45	NS	2.25 ± 0.20	2.40 ± 0.56	NS
Posterior mandible height (mm)	3.87 ± 0.10	4.10 ± 0.30	NS	4.98 ± 0.29	4.37 ± 0.056	NS

Supplementary figure S2

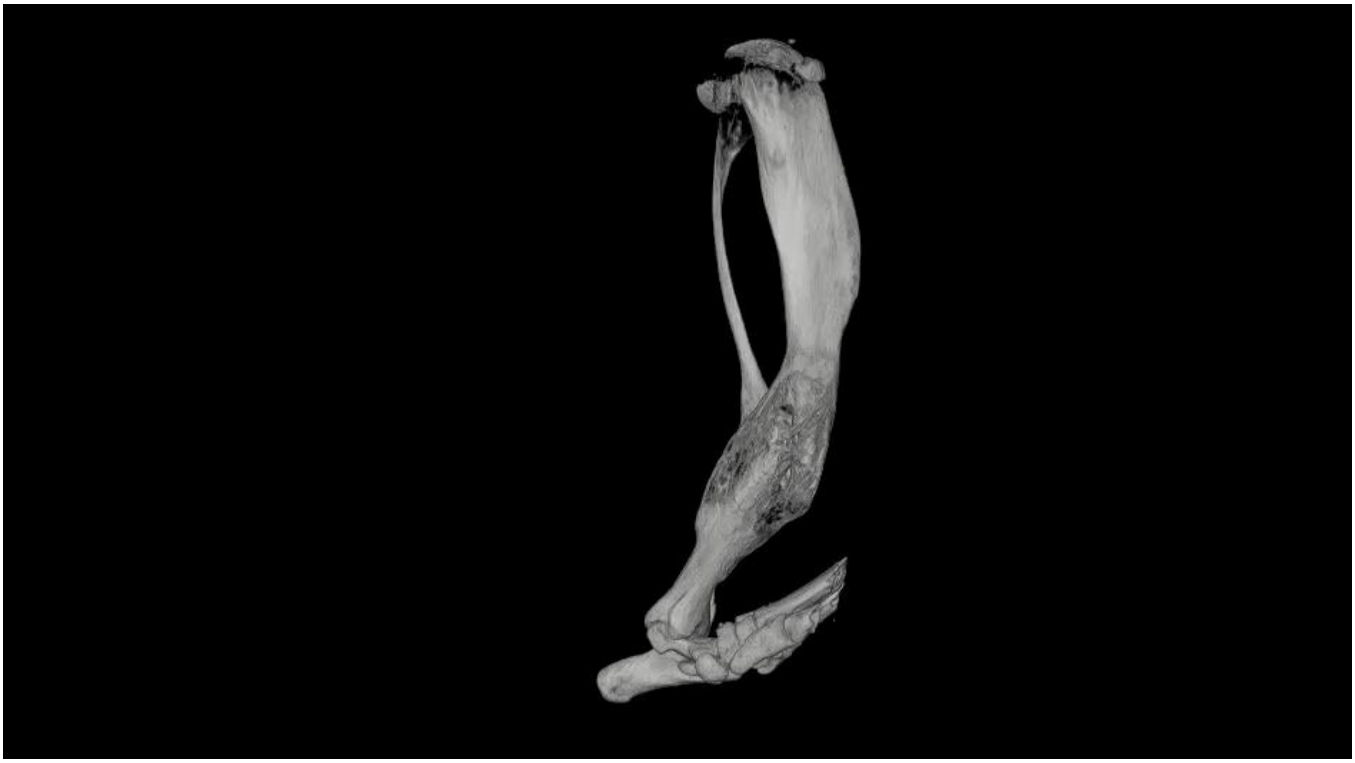

Supplementary figure S2. Abnormalities in cortical bone at the tibiofibular junction in male $_{\text{Ocn}}$VEGFKO. Video showing a rotation of the whole tibia of a 16 week old male mouse. Thinning is observed at the epiphysis and the tibiofibular junction (see attached video clip).

Supplementary table S3: Individual threshold values defining the separation between 16 week old osteocyte lacunae and intracortical canals for each animal.

	Female ID	Threshold (µm³)	Male ID	Threshold (µm³)
WT	1	1922	1	1922
	2	1785	2	1648
	3	1648	3	1648
OcnVEGFKO	1	6591	1	6042
	2	2472	2	9337
	3	1648	3	7140

Supplementary figure S3

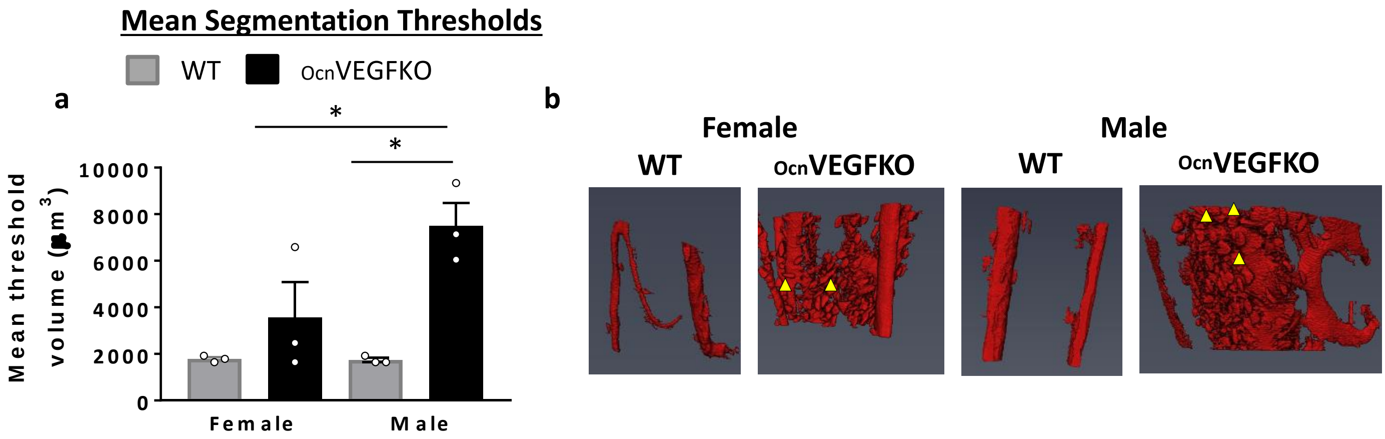

Supplementary figure S3. Sexual dimorphism in osteocyte/intracortical canal separation thresholds. (a) Average separation threshold is just below the volume of the smallest intracortical canal for each individual bone (values and error bars indicate mean value ± SEM, n = 3 females and n=3 males, $p<0.05$* using t-test). (b) 3D rendering of porosity above these threshold values using Aviso 9 (FEI, Hillsboro, OR, USA) gives an idea of the structures segmented in this larger fraction. Where WT canals were smooth in appearance, OcnVEGFKO canals were abnormal in shape due to the attachment of osteocyte lacunae (yellow arrows) to their outer surface.

Supplementary table S4: Proportions above/below the threshold of small osteocyte lacunae, for each individual animal.

Sex	Mutation	Repeat	Num.High	Num.Low	Num.Total	Low.Propor	High.Propor
Female	WT	1	7348	1175	8523	0.1378623	0.8621377
Female	WT	2	9391	721	10112	0.0713014	0.9286986
Female	WT	3	8416	733	9149	0.080118	0.919882
Female	OcnVEGFKO	1	7393	1465	8858	0.1653872	0.8346128
Female	OcnVEGFKO	2	7397	1504	8901	0.1689698	0.8310302
Female	OcnVEGFKO	3	8215	1405	9620	0.1460499	0.8539501
Male	WT	1	10522	1417	11939	0.1186867	0.8813133
Male	WT	2	9709	1182	10891	0.10853	0.89147
Male	WT	3	7519	1495	9014	0.1658531	0.8341469
Male	OcnVEGFKO	1	7526	7466	14992	0.4979989	0.5020011
Male	OcnVEGFKO	2	8953	6213	15166	0.4096664	0.5903336
Male	OcnVEGFKO	3	7693	9775	17468	0.5595947	0.4404053

Supplementary table S5: Quantitative morphometric measures for intracortical vascular canals and osteocyte lacunae of 4 and 16-week-old WT and $_{Ocn}$VEGFKO mice. Intracortical canals and osteocyte lacunae have been defined using individual thresholds for each animal. Quantification is performed using 300 slices per dataset.

	4 week old						16 week old					
	Female			Male			Female			Male		
	WT	$_{Ocn}$VEGFKO	p	WT	$_{Ocn}$VEGFKO	p	WT	$_{Ocn}$VEGFKO	p	WT	$_{Ocn}$VEGFKO	p
Lc.V (μm^3)	421±52	403±26	NS	425±23	425±34	NS	363±24	423±33	p<0.05	423±21	421±19	NS
Ca.V (μm^3)	21023±8262	62560±28310	NS	20189±4737	79296±19376	p<0.05	16794±8598	35362±14434	NS	11144±3749	862208±374663	P<0.01
Lc.Dm (μm)	4.32±0.48	4.21±0.70	NS	4.49±0.59	3.99±0.6	NS	3.27±0.20	3.69±0.10	p<0.05	3.78±0.09	3.25±0.05	p<0.001
Ca.Dm (μm)	6.77±1.43	6.49±1.59	NS	6.90±0.94	7.02±1.88	NS	5.90±1.13	6.63±0.94	NS	5.60±0.52	6.17±0.48	NS
Number of Osteocyte Lacunae (# per mm3)	74265±7267	88399±10108	NS	80104±8404	88219±12472	NS	66339±857	73645±1472	p<0.05	64364±2238	70130±3822	NS
Number of Blood Vessel Canals (# per mm3)	1379±538	1001±435	NS	1050±254	614±235	NS	682±390	625±228	NS	819±103	170±62	p<0.01

Supplementary figure S4

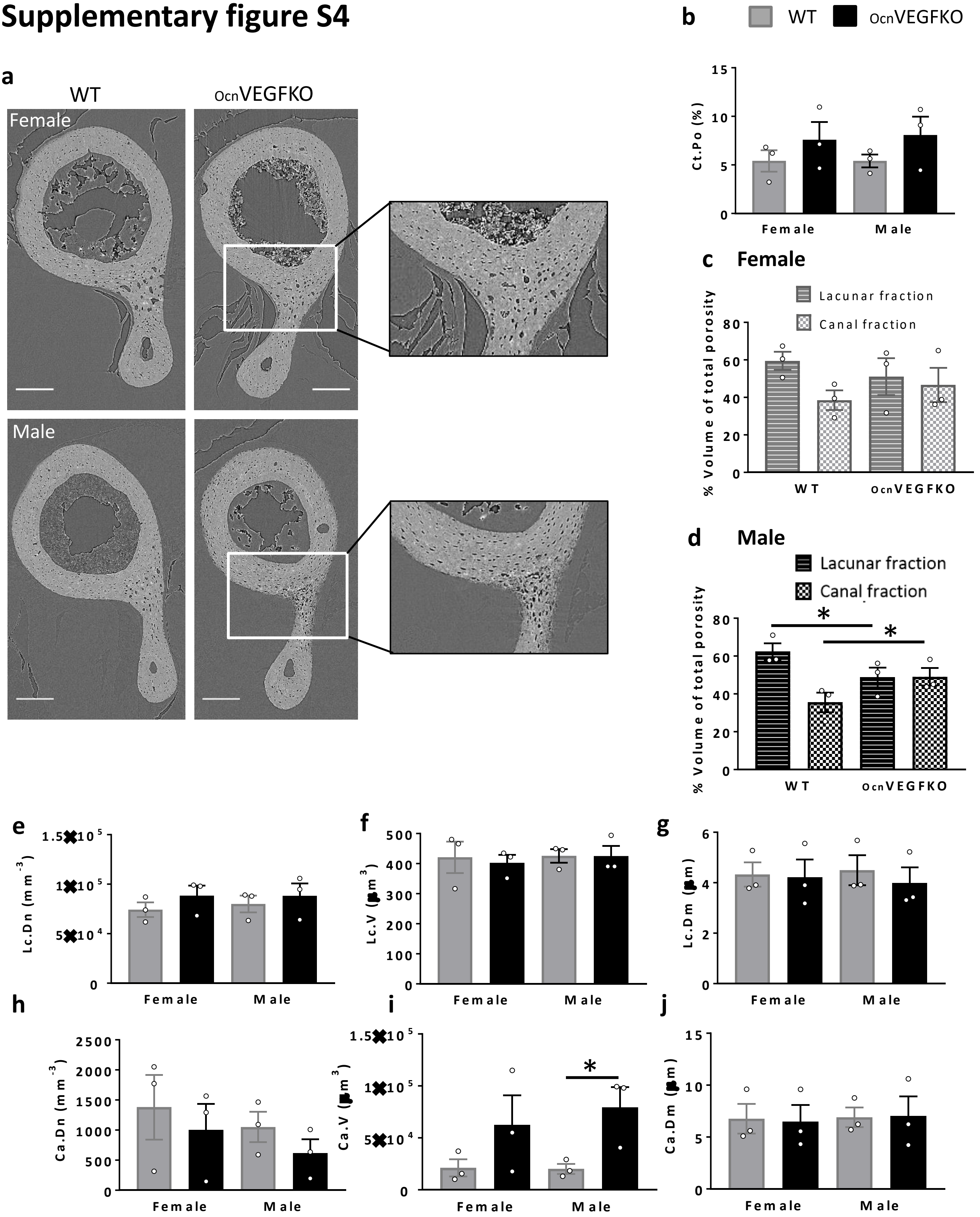

Supplementary figure S4. Distinction of bone porosity components is compromised following OcnVEGFKO in 4 week old mice. High resolution, synchrotron X-ray computed tomography (SRCT; g; 0.65µm voxel size) slices from 4 week old female and male WT and OcnVEGFKO mice (a) revealed poorly mineralised areas of cortical bone at the tibiofibular junction (white square). The % cortical porosity was not significantly changed in both males and females following OcnVEGFKO (b). In female OcnVEGFKO there was no significant difference in the % porosity constituting the intracortical canal fraction versus WT (c). In male OcnVEGFKO, % pores constituting the intracortical canal fraction was increased versus WT (d). Measurements taken of regularly sized osteocyte lacunae show no significant differences in lacunae number density (Lc.Dn; e), mean lacunar volume Lc.V; f) or (Lc.Dm; g) in males or females following knockout of VEGF versus WT. Number of intracortical canals greater than the lacunar threshold (see methods) was not significantly changed in OcnVEGFKO (Ca.Dn; h), however there was a significant change in mean canal volume (Ca.V; i) versus WT in male OcnVEGFKO. Mean canal diameter was not significantly altered in male OcnVEGFKO (Ca.Dm; j). Error bars indicate mean value ± SEM, n=3 females and 3 males from 2 individual litters $p<0.05$*, using two way ANOVA.

Supplementary figure S5

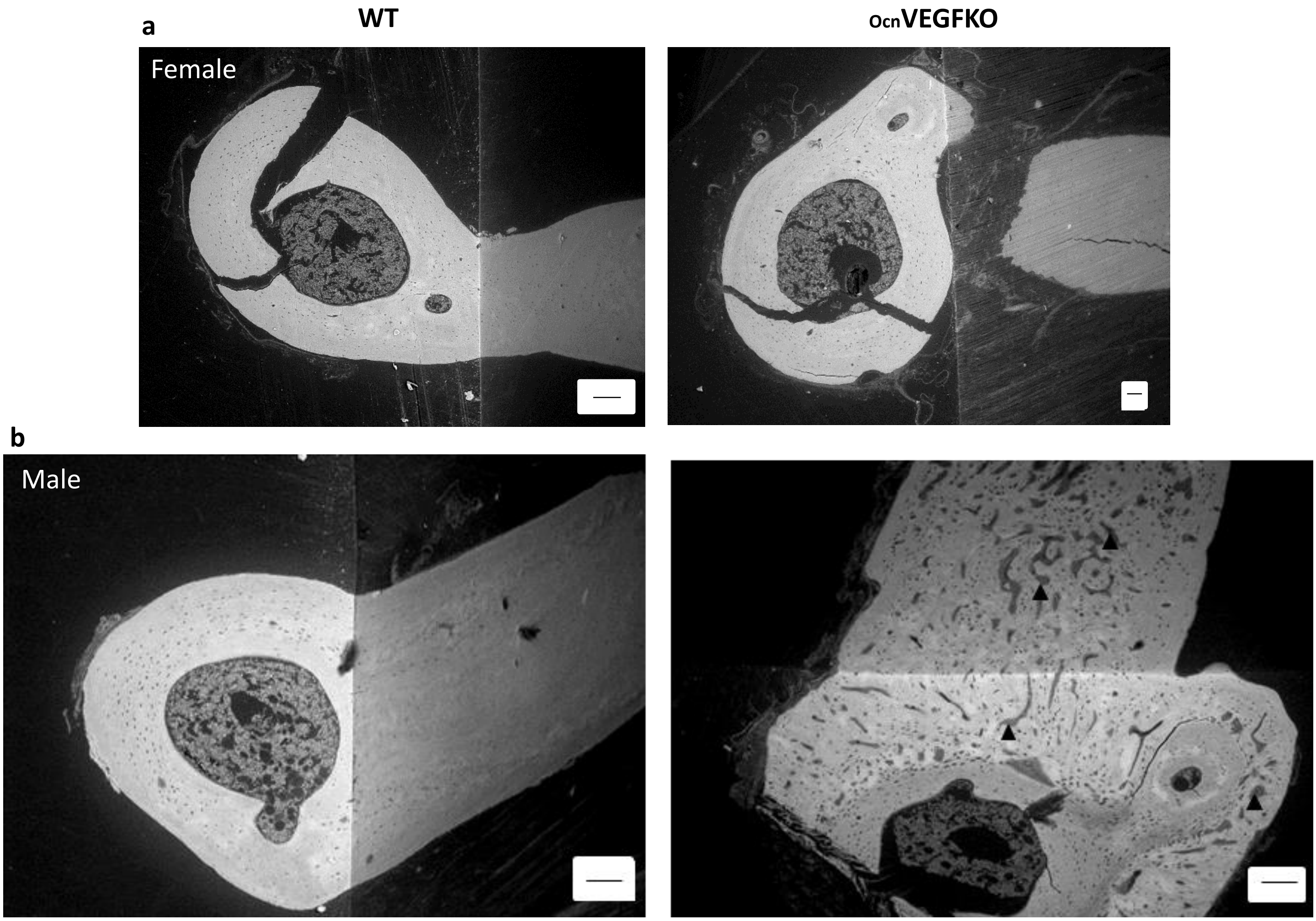

Supplementary figure S5. Deletion of VEGF in males causes increased osteoid and porosity along the bone length. Minimal differences in the morphology of the porosity and presence of osteoid are seen in WT versus OcnVEGFKO female longitudinal BSE SEM images of tibia which have been stained with iodine vapour (a). Following OcnVEGFKO in males, an increase in osteoid surrounding blood vessels (black arrows) and osteocytes, along with irregularly shaped porosity is visible along the length of the bone, in comparison to WT SEM images (b). Scale bar = 100µm.

Supplementary figure S6

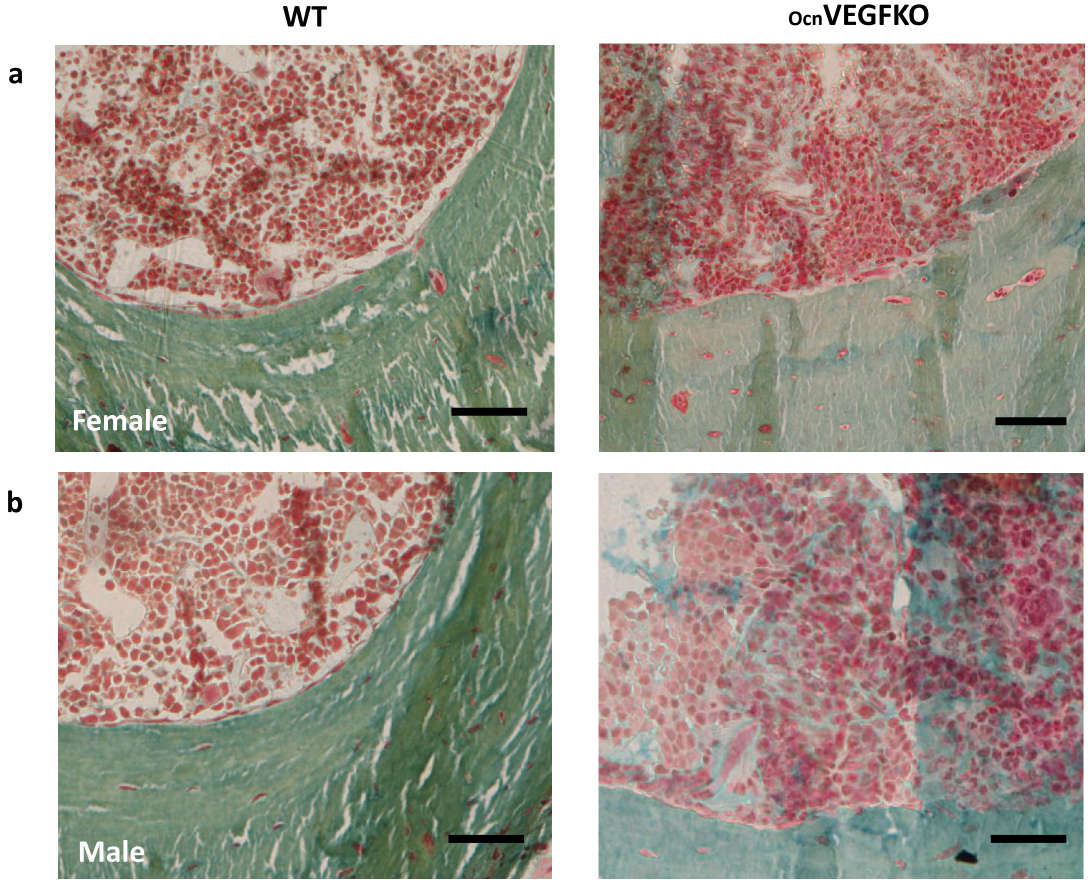

Supplementary figure S6. Endosteal remodelling in female (a) and male (b) OcnVEGFKO and WT animals (16 weeks). Scale = 100µm.

Supplementary figure S7

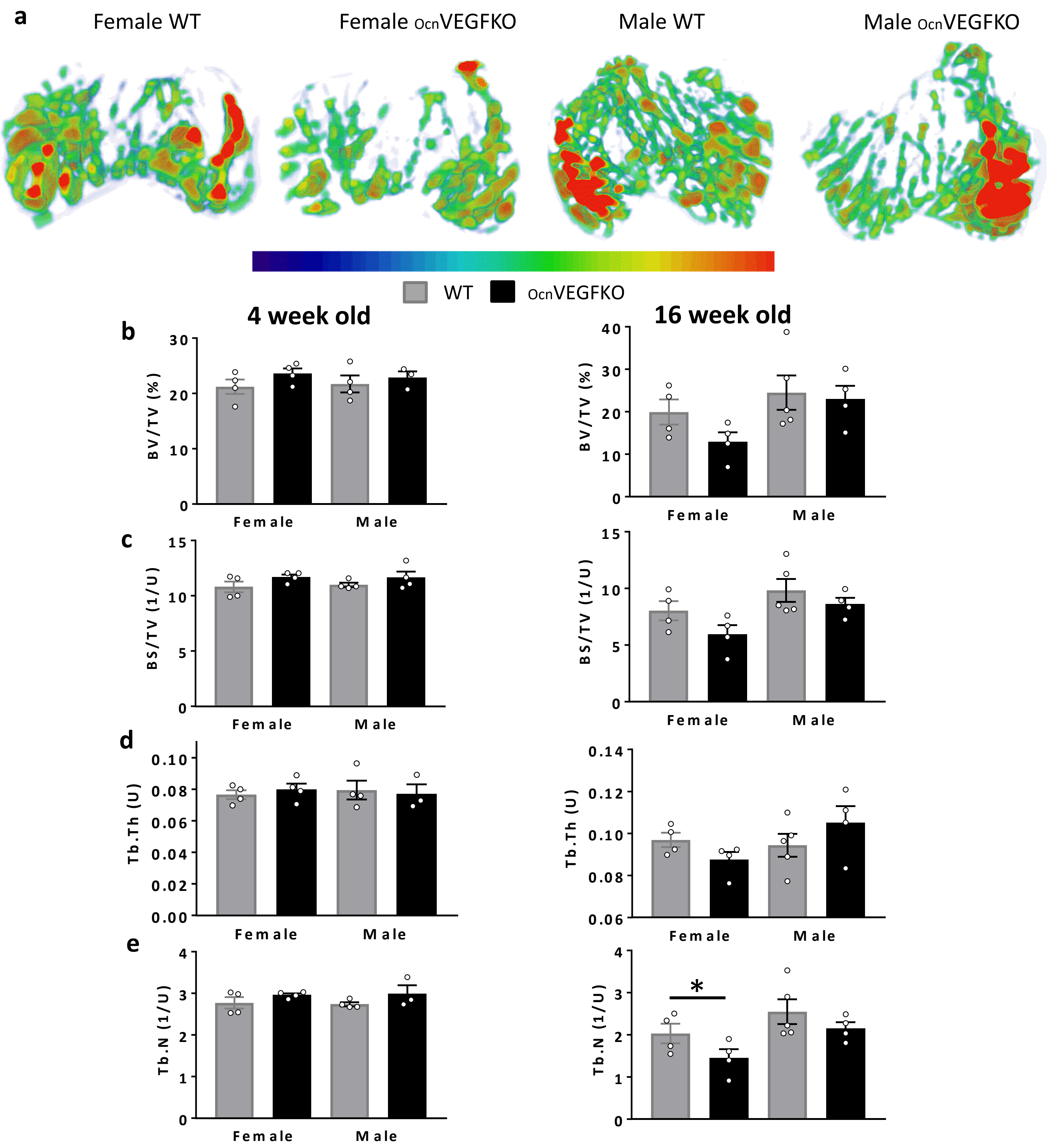

Supplementary figure S7. Minimal changes in trabecular measurements in 4 and 16 week male and female mice following OcnVEGFKO. Trabecular thickness for 16 week old male, female, WT and OcnVEGFKO tibiae is displayed as a heatmap with blue representing the thinnest trabeculae and red representing the thickest trabeculae. For both age groups, no differences in % bone volume (b; BV/TV), bone surface area (c; BS/TV) and trabecular thickness (d; Tb.Th) were observed. A significant decrease in trabecular number was identified in 16 week old female animals only, following OcnVEGFKO (e). Error bars indicate mean value ± SEM, $p<0.05$*, using two way ANOVA. 4 week; n=4 female WT, female OcnVEGFKO, male WT and n=3 male OcnVEGFKO. 16 week; n=4 female WT, female OcnVEGFKO and male OcnVEGFKO and n=5 male WT.

Supplementary figure S8

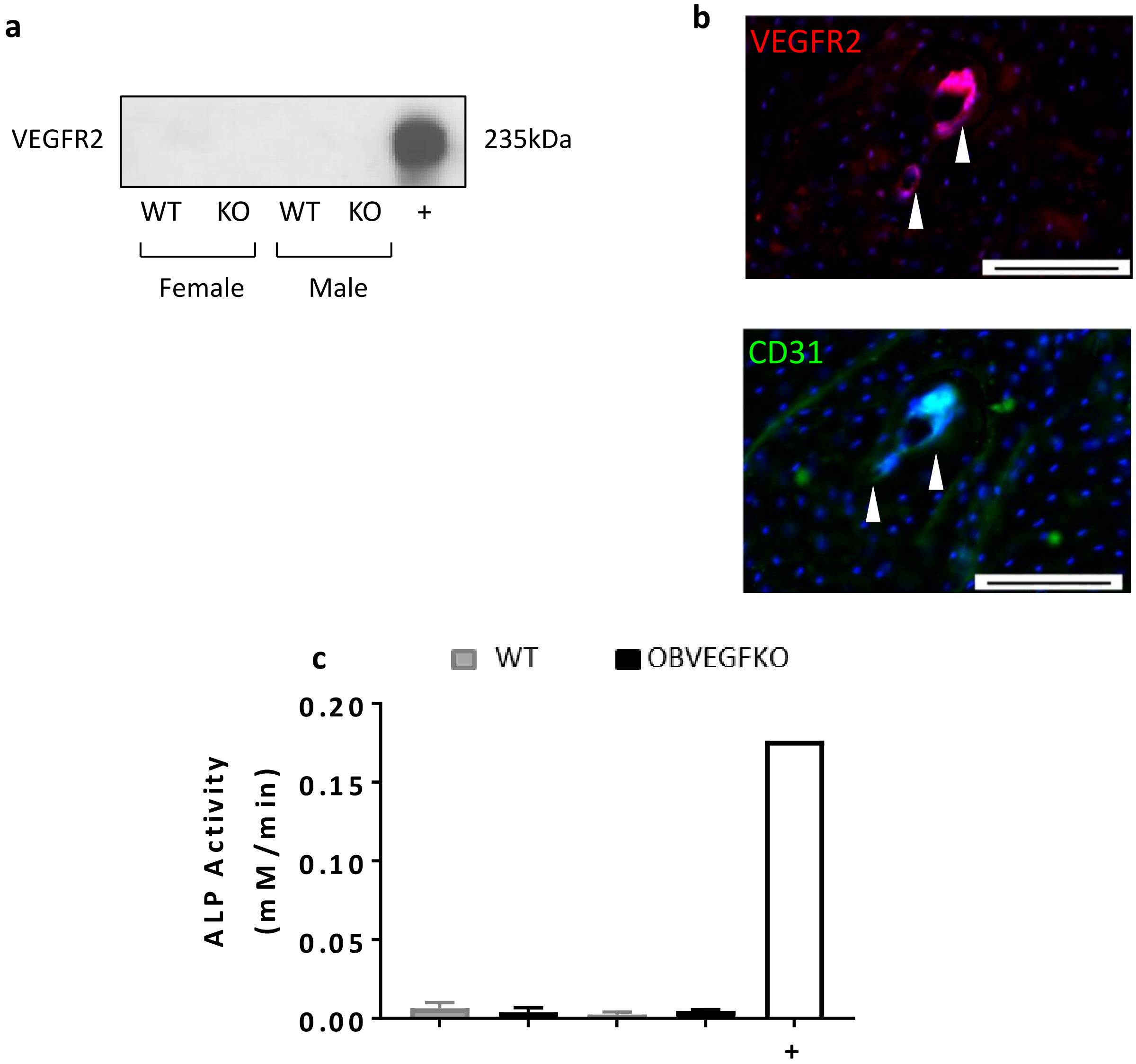

Supplementary figure S8. Lack of VEGFR2 expression in osteoblasts. Western blot confirms the lack of expression of VEGFR2 in osteoblast cell lysates with or without VEGF (a). Endothelial cell lysate is used here as a positive control. Immunohistochemistry of cryo sections staining for both CD31 and VEGFR2 confirmed the co-localisation of VEGFR2 (b) with the bone vasculature (white arrows; CD31+ endothelial cells). qPCR using cDNA reverse transcribed from both OB cell lysates (n=3) and a MBMEC cell lysate as a positive control (+), further confirmed the low levels of VEGFR2 expression in both male and female OBs (c).

Supplementary figure S9

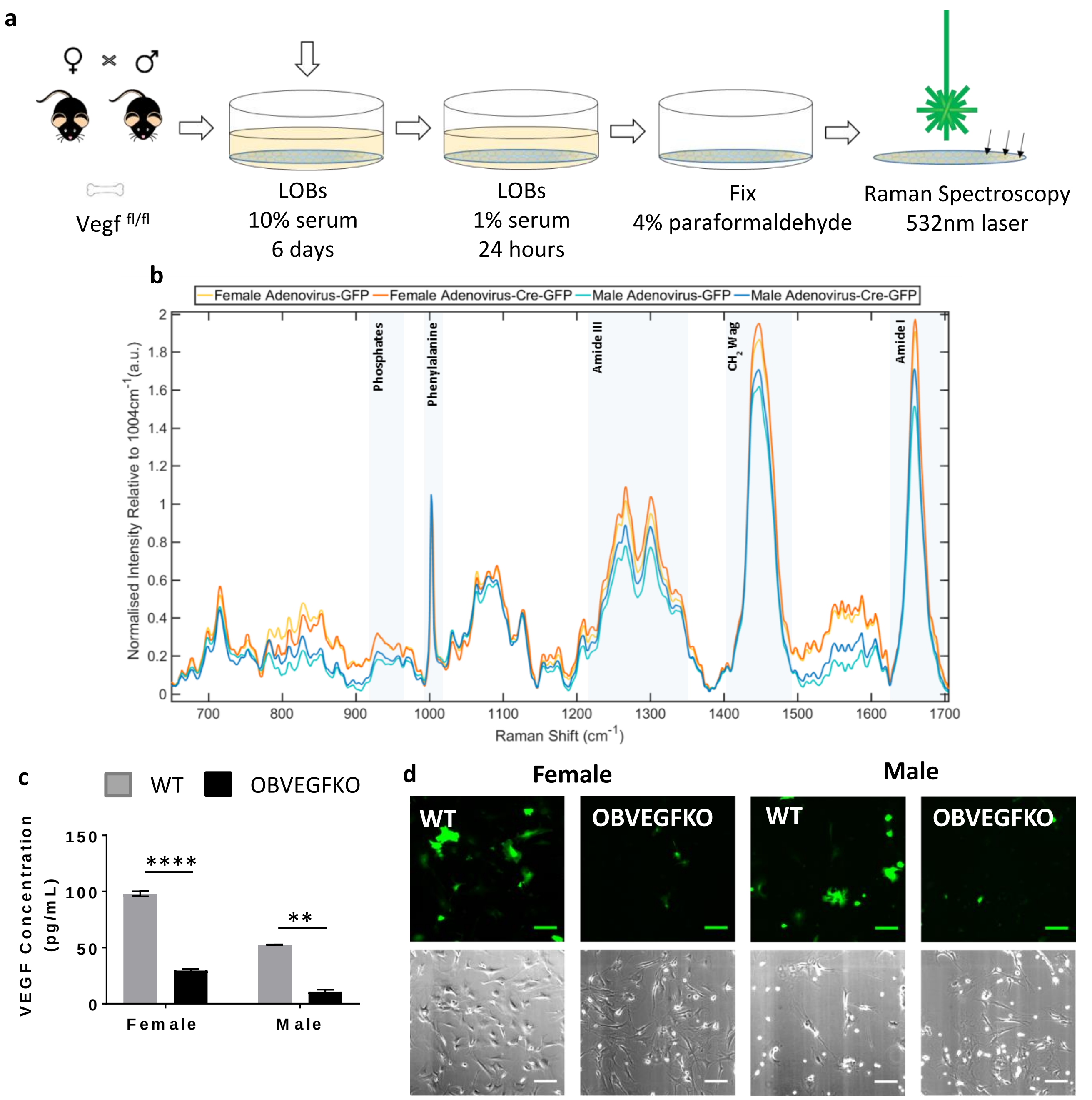

Supplementary figure S9. Deletion of VEGF in female and male osteoblasts can be detected by Raman spectroscopy. Schematic of the experimental methodology is shown (a). LOBs extracted from the long bones of 4 day old male and female VEGF $^{fl/fl}$ mice AND cells were infected with adenovirus-GFP or adenovirus-Cre-GFP for 6 days in 10% FBS media at a MOI of 100, before being stepped down for 24 hours in 1% FBS media on quartz coverslips. Following fixation with 4% paraformaldehyde, Raman spectra were collected over the cytoplasm of 10 cells per condition at 5 separate points. Class means of Raman spectra normalised to the Raman peak of phenylalanine (1004cm^{-1}) are displayed (b). Spectral differences are noted in the phosphate region (948cm^{-1} to 970cm^{-1}), amide III (1242cm^{-1}), CH_2 deformation (1450cm^{-1}) and amide I (1660cm^{-1}). Conditioned media were collected from osteoblast cultures to confirm VEGF deletion using an VEGF ELISA (c). Data represents mean VEGF concentration ± SEM, p<0.01**, p<0.0001**** using t-test. Representative fluorescence and phase contrast microscopy images of male and female LOB cultures confirmed successful infection with adenovirus-GFP and adenovirus-Cre-GFP (d). Scale bar = 100µm.

Supplementary figure S10

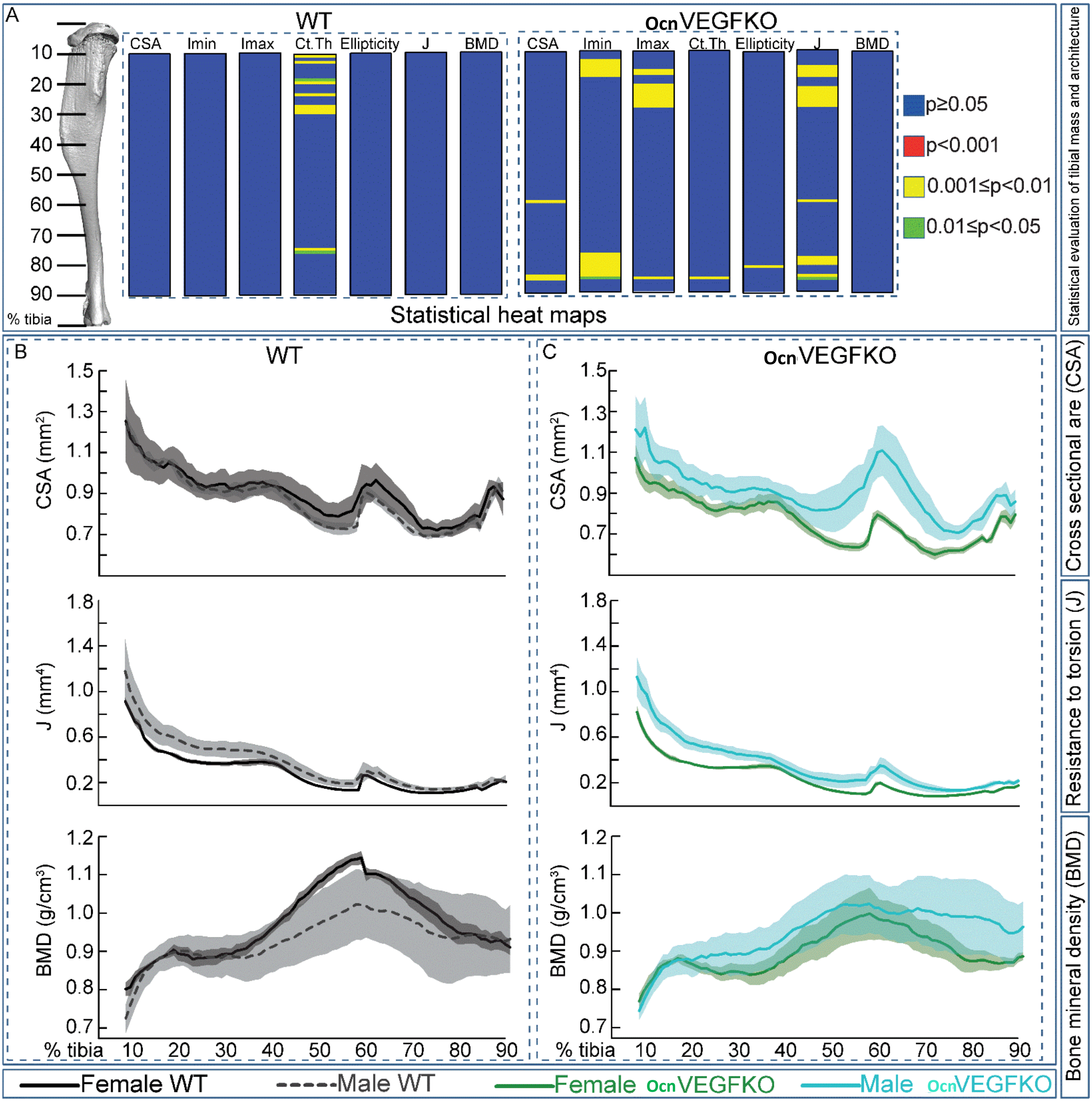

Supplementary figure S10. Sex differences evident along the tibial length in 16 week old WT and OcnVEGFKO bones. Graphical heat map summarises statistical differences between female versus male subjects within WT and OcnVEGFKO groups at specific matched locations along the tibial length (a). Red p≤0.0001, yellow p≤0.001-0.01, green p≤0.01-0.05 and blue ≥0.05. Line graphs represent means for WT (b) and OcnVEGFKO (c) females versus males ± SEM (n=4 males and females from individual litters). Statistics performed using ANOVA.

Supplementary figure S11

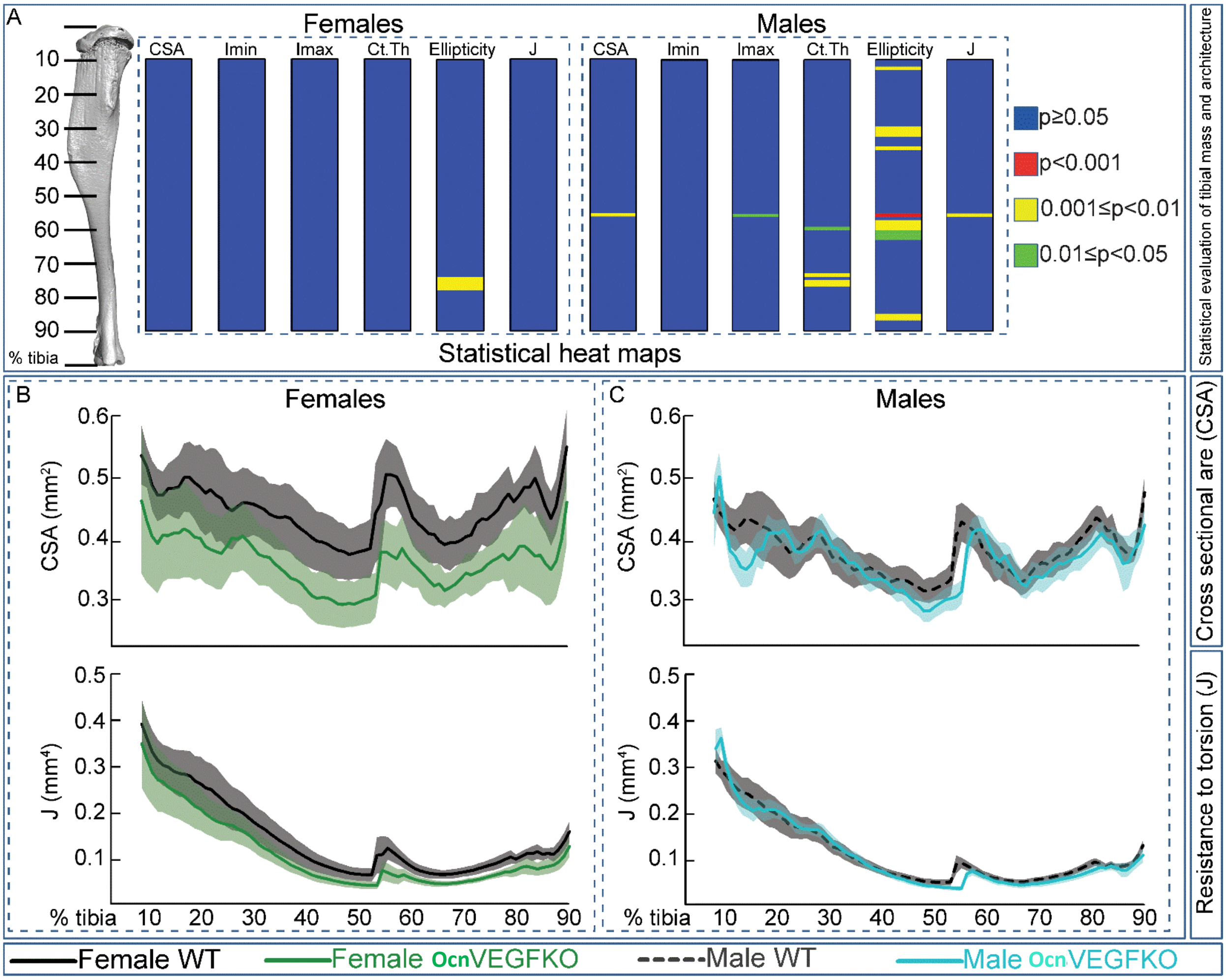

Supplementary figure S11. Geometrical alterations present in 4 week old male tibia following OcnVEGFKO**.** Graphical heat map summarises statistical differences between OcnVEGFKO versus WT mice within female and male groups at specific matched locations along the tibial length (a). Red $p \leq 0.0001$, yellow $p \leq 0.001$-0.01, green $p \leq 0.01$-0.05 and blue ≥ 0.05. Line graphs represent means for female (b) and male (c) WT versus OcnVEGFKO ± SEM (n=4 males and females from 2 individual litters). Statistics performed using ANOVA.
