# Supplementary material for: Regulation of the Bone Vascular Network is Sexually Dimorphic
Source: J Bone Miner Res. 2019 Oct 9;34(11):2117–32. doi: 10.1002/jbmr.3825 (PMC6899569; doi:10.1002/jbmr.3825)
Supplement: Supplementary file 3 — Supporting information. [file JBMR-34-2117-s003.doc]

**Supplementary Methods 1**

High resolution micro-CT analysis; determining the osteocyte lacunae and blood vessel canal separation threshold

Typically, a global size threshold is used for the separation of lacunae and intracortical canals based on particle size^1-3^. However, when a global size threshold of 1510μm^3^ was used to separate lacunae and intracortical canals based on porosity distributions in WT animals, a proportion of large osteocyte lacunae was detected in the vascular fraction in OcnVEGFKO bones. As a result, individual size thresholds were used to separate lacunae and intracortical canals and set just below the volume of the smallest intracortical canal (Supplementary table S2).

Correlating with previous studies ^3,4^, particles below 27.4 μm^3^ in size were defined as noise. Particles volumetrically larger than noise, but smaller than the individual threshold values which separate the lacunae and the canals (Supplementary table S2) were categorised as osteocyte lacunae. The mean separation threshold used for OcnVEGFKO males was significantly greater than the thresholds used for WT controls and OcnVEGFKO females (Supplementary figure S3a). Surprisingly in OcnVEGFKO animals, some osteocyte lacunae contributed to the canal volume fraction due to their intimate proximity (i.e. direct connection) to the intracortical canals (Supplementary figure S3b) and therefore could not be separated. It should be noted that subsequent measures of the canal volume fraction in the OcnVEGFKO male and female animals include these ‘attached’ lacunar volumes

Supplementary references

1 Mosey, H. *et al.* Sost Deficiency does not Alter Bone's Lacunar or Vascular Porosity in Mice. *Front Mater* **4**, 27, doi:10.3389/fmats.2017.00027 (2017).

2 Javaheri, B. *et al.* Phospho1 deficiency transiently modifies bone architecture yet produces consistent modification in osteocyte differentiation and vascular porosity with ageing. *Bone* **81**, 277-291, doi:10.1016/j.bone.2015.07.035 (2015).

3 Carriero, A. *et al.* Altered lacunar and vascular porosity in osteogenesis imperfecta mouse bone as revealed by synchrotron tomography contributes to bone fragility. *Bone* **61**, 116-124, doi:10.1016/j.bone.2013.12.020 (2014).

4 McCreadie, B. R., Hollister, S. J., Schaffler, M. B. & Goldstein, S. A. Osteocyte lacuna size and shape in women with and without osteoporotic fracture. *J Biomech* **37**, 563-572, doi:10.1016/S0021-9290(03)00287-2 (2004).
